# *Pseudomonas aeruginosa* maintains an inducible array of novel and diverse prophages over lengthy persistence in cystic fibrosis lungs

**DOI:** 10.1093/femsle/fnaf017

**Published:** 2025-01-31

**Authors:** Ifigeneia Kyrkou, Jennifer Bartell, Ana Lechuga, Cédric Lood, Rasmus L Marvig, Rob Lavigne, Søren Molin, Helle Krogh Johansen

**Affiliations:** Department of Veterinary and Animal Sciences, Food Safety and Zoonosis, University of Copenhagen, 1870 Frederiksberg, Denmark; The Novo Nordisk Foundation Center for Biosustainability, Technical University of Denmark, 2800 Kgs. Lyngby, Denmark; Department of Clinical Microbiology, Rigshospitalet, 2100 Copenhagen, Denmark; Laboratory of Gene Technology, Department of Biosystems, KU Leuven, 3001 Heverlee, Belgium; Laboratory of Gene Technology, Department of Biosystems, KU Leuven, 3001 Heverlee, Belgium; Laboratory of Computational Systems Biology, Department of Microbial and Molecular Systems, KU Leuven, 3001 Heverlee, Belgium; Center for Genomic Medicine, Rigshospitalet, 2100 Copenhagen, Denmark; Laboratory of Gene Technology, Department of Biosystems, KU Leuven, 3001 Heverlee, Belgium; The Novo Nordisk Foundation Center for Biosustainability, Technical University of Denmark, 2800 Kgs. Lyngby, Denmark; The Novo Nordisk Foundation Center for Biosustainability, Technical University of Denmark, 2800 Kgs. Lyngby, Denmark; Department of Clinical Microbiology, Rigshospitalet, 2100 Copenhagen, Denmark; Department of Clinical Medicine, University of Copenhagen, 2100 Copenhagen, Denmark

**Keywords:** phages, genomics, persistence, ecology, *P. aeruginosa*, cystic fibrosis

## Abstract

*Pseudomonas aeruginosa* has increasing clinical relevance and commonly occupies the cystic fibrosis (CF) airways. Its ability to colonize and persist in diverse niches is attributed to its large accessory genome, where prophages represent a common feature and may contribute to its fitness and persistence. We focused on the CF airways niche and used 197 longitudinal isolates from 12 patients persistently infected by *P. aeruginosa*. We computationally predicted intact prophages for each longitudinal group and scored their long-term persistence. We then confirmed prophage inducibility and mapped their location in the host chromosome with lysate sequencing. Using comparative genomics, we evaluated prophage genomic diversity, long-term persistence, and level of genomic maintenance. Our findings support previous findings that most *P. aeruginosa* genomes harbour prophages some of which can self-induce, and that a common CF-treating antibiotic, ciprofloxacin, can induce prophages. Induced prophage genomes displayed high diversity and even genomic novelty. Finally, all induced prophages persisted long-term with their genomes avoiding gene loss and degradation over 4 years of host replication in the stressful CF airways niche. This and our detection of phage genes, which contribute to host competitiveness and adaptation, lends support to our hypothesis that the vast majority of prophages detected as intact and inducible in this study facilitated their host fitness and persistence.

## Teaser

Clinical *Pseudomonas aeruginosa* genomes contain an array of intact, inducible prophages, which are genomically highly diverse sometimes novel, and persist long-term in their niche with their genomes virtually unchanged.

## Introduction

Bacteriophages (phages) are viruses of bacteria that often show high infection specificity. While phages with a strictly lytic lifestyle (virulent) rapidly kill their bacterial host, phages with a lysogenic lifestyle (temperate) can also integrate into the bacterial genome, in a form termed prophage. Temperate phage integration incurs a metabolic burden to the host bacterium (hereafter host). However, this burden can be outweighed by certain fitness benefits via the prophage (a) increasing host fitness via beneficial gene(-s) (morons), (b) offering immunity to infection by related phages (i.e. superinfection exclusion), (c) protecting the host from phagocytosis of predators or the human immune system, (d) beneficially regulating the host phenotype or driving host fitness by altering host gene expression upon its genomic integration or excision within an open reading frame (active lysogeny and transposition, respectively), and/or (e) reverting to the lytic lifestyle (i.e. inducing) due to the stress incurred to the host by competing strains within a mixed population, where phage-susceptible competitors are killed in higher proportion to the host (Meltz Steinberg and Levin 2007, Rabinovich et al. 2012, Fortier 2017, Secor et al. 2017, Argov et al. 2019, Marshall et al. 2021). The latter case appears to be influenced by the presence of other advantageous fitness traits, which in addition to the prophage collectively enhance the host’s dominance over its competitors (Marshall et al. 2021). Prophages occur frequently in the genomes of many human pathogenic bacteria, including *Acinetobacter baumannii, Klebsiella pneumoniae, Escherichia coli*, and *Staphylococcus aureus* (Ingmer et al. 2019, de Sousa et al. 2020, Loh et al. 2020). It is therefore theorized that prophages influence infection processes of a pathogen either by controlling its population size or by modifying its genomic content and/or phenotype (Lawrence et al. 2019).

Bacteria that establish persistent infections in human lungs show a broad diversity of prophages (Willner et al. 2009). An example of a bacterium that can persistently infect the human airways is *Pseudomonas aeruginosa. P. aeruginosa* is associated with severe morbidity and mortality in cystic fibrosis (CF) patients. Its persistent infections cause chronic lung inflammation and can last for >30 years (Folkesson et al. 2012), requiring continuous antibiotic treatment, and undermining life quality due to impaired lung function (Rajan and Saiman 2002). While *P. aeruginosa* opportunistically colonizes the human body, it also occupies a plethora of ecological niches, from soil and water to plants, insects, and animals (Kung et al. 2010). The ability of this bacterium to adapt to various niches may be attributed to mobile genetic elements (Kung et al. 2010), especially considering the bacterium’s mediocre capacity for natural transformation (Nolan et al. 2020). Regardless of niche, it is common for a *P. aeruginosa* strain to be lysogenized by one or two temperate phages (Johnson et al. 2022), which are suggested to be important drivers of this bacterium’s genomic plasticity (Shen et al. 2006).

In recent years, a renewed interest in temperate phages of *P. aeruginosa* has mainly targeted their role in shaping host virulence, often overlooking other impacts that these may have on host fitness and survival. Related studies identified a number of prophage genes that contribute to host virulence (Schroven et al. 2021). The case of the Liverpool Epidemic Strain (LES) constitutes a notable example of how prophages can influence host fitness; three of its five intact prophages, LESφ2–3-5, were found essential for LES colonization in a rat lung infection model (Winstanley et al. 2009). Furthermore, strain PAO1 lysogens of prophages LESφ2–3-4 increased competitiveness against nonlysogenic PAO1 in the same rat model (Davies et al. 2016a). In a recent study (Marshall et al. 2021), the crucial role of prophages in mediating fitness and persistence of specific *P. aeruginosa* clones was clearly demonstrated using a mixed *P. aeruginosa* population to infect a porcine wound model. Two prophages, a F116-like and a JDB24-like, released from transient competitor strains, drove the emergence of strains with the hyperbiofilm-forming ‘novel rugose small colony variant’ phenotype that could persist over other ancestral strains under wound infection settings.

Here, we investigated the abundance, activity, diversity, and long-term maintenance of intact prophages that reside in the genomes of bacterial isolates from distinct clonal lineages of *P. aeruginosa* evolving in CF airways. We do this by selecting 12 comparable CF airway niches by defined criteria, bioinformatically screening *P. aeruginosa* isolates from these 12 niches to identify frequent and potentially intact prophages, choosing 12 representative isolates from each niche to experimentally confirm prophage activity, and interpreting findings within the complex evolutionary context that shapes persistent infections. Other studies have partially addressed this topic by either focusing on a single, genetically distinct clonal lineage (or clone type, CT) such as the highly transmissible LES or by assessing exclusively *in silico* the completeness and activity of potential prophage regions. The present study is the first to validate findings of *in silico* genome-based predictions on prophage activity with *in vitro* activity experiments using a wide range of CTs. Moreover, this study is unique in assessing the long-term maintenance of *P. aeruginosa* prophages from 12 parallel niches (individual CF airways) in high temporal resolution. With these, we aim not only to enrich current knowledge on *Pseudomonas* phage ecology, evolution and genomics but also to interrogate the potential contributing role of prophages for the persistence of *P. aeruginosa* in the CF airways niche.

## Materials and methods

### 2.1. Isolate collection and culture conditions

**Figure 1.**
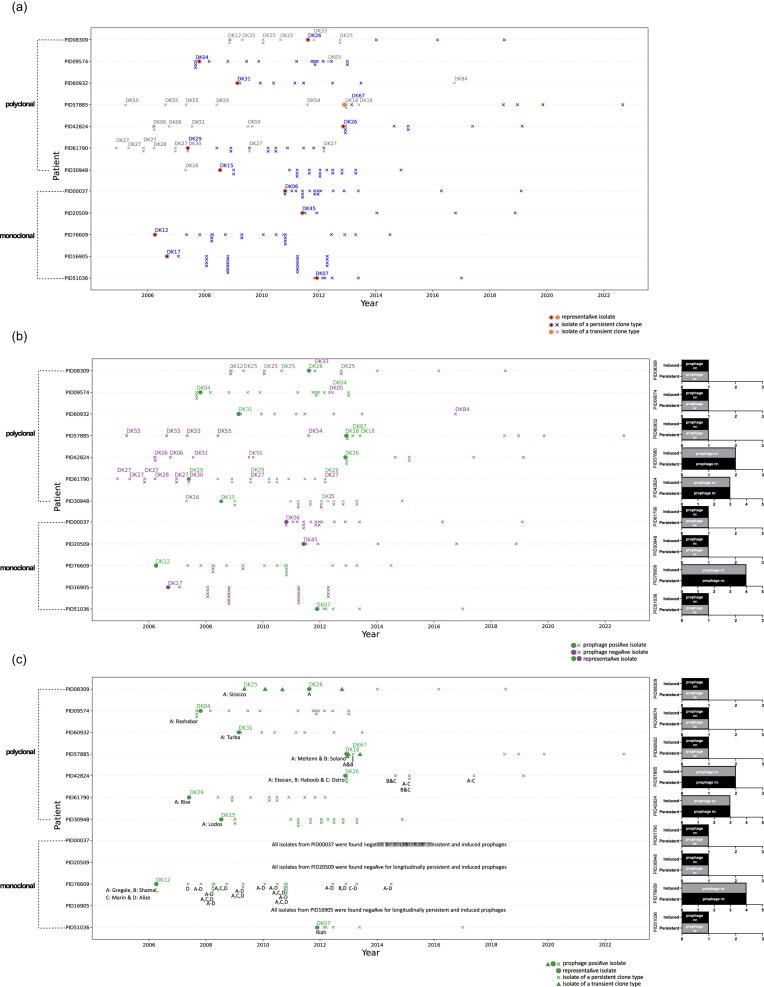
(a) Overview of the collection of 197 longitudinal isolates from this study, originating from the airways of 12 CF patients (12 PID#), who were persistently infected with P. aeruginosa between 2006 and 2022. The genomes of all 197 isolates (see main text for definition of the term ‘isolate’) were scanned *in silico* to detect intact prophage elements. In this panel (panel A), isolates of the collection are represented by dot and cross markers and plotted according to the patient they were isolated from (*y*-axis) and their date of isolation (*x*-axis; only year is indicated to maintain patient data privacy). Within each patient’s longitudinal-isolate history row (drawn as a horizontal line), orange-haloed dots are used to mark the 12 representative isolates, one per patient, that are later used to perform *in vitro* prophage inductions. These are: PID08309 (isolate 188), PID09574 (isolate 37), PID60932 (isolate F038), PID57885 (isolate LRJ32), PID42824 (isolate 382), PID61790 (isolate 199), PID30948 (isolate 135), PID00037 (isolate F056), PID20509 (isolate F023), PID76609 (isolate F002), PID16905 (isolate F004), and PID51036 (isolate 20). On the left side of panel A, patients are clustered into two groups; a polyclonal group that represents patients infected by multiple *P. aeruginosa* CTs (see main text for definition of the term CT), and a monoclonal group that comprises patients infected by a single CT of *P. aeruginosa*. All distinct CTs are registered using ID numbers that begin with ‘DK’. Only one persistent CT exists per patient row, with the term ‘persistent’ defining the CT that was longitudinally retraced the longest and for a period of least 4 years within that patient. The ID number of each persistent CT appears in blue font and labels only the first occurrence of the persistent CT within that patient, i.e. the first isolate of the persistent CT. The isolates of the persistent CT are marked using either a blue, orange-haloed dot (representative isolate) or a blue cross. Per patient row, all nonpersistent CT isolates belong to one or more transient CTs. Isolates of transient CTs (i.e. of CTs that are retraced for a period shorter than that of the persistent CT in a given patient) are represented by a grey cross or grey, orange-haloed dot (PID57885 is the only patient with representative isolate from a transient CT). The CT ID number of each transient CT isolate is consistently registered above its marker in grey font. (b) Copy of plot from panel a. In this version (panel B), green colour marks all isolates positive for at least one detected as longitudinally persistent and induced prophage. Purple colour marks all isolates that were negative for such prophages. Details on which specific prophage each isolate was positive for are presented in panel C. On the right side of panel B, smaller plots display the part of induced prophages, which were found long-term in the genome of the persistent CT (bottom bar; indicated as ‘persistent’) within a green-isolate patient, as compared to the sum of induced prophages within the same patient (top bar; indicated as ‘induced’). (c) Copy of plot from panel B. This version complements panel B, in that it labels the specific prophages that were detected as longitudinally persistent and induced per green isolate of panel B. This information, along with details on which predicted as intact but uninduced prophages were identified per longitudinal isolate, is also listed in Table S1. Isolates marked as purple in panel B are removed to retain only the green, prophage positive isolates, which are represented by cross, dot, and pyramid markers. Marker symbols are used as before except pyramids, which are introduced to mark isolates that belonged to transient CTs. Prophages that were detected as longitudinally persistent and induced within a given patient row are abbreviated using letters from A to D and are registered immediately below the first green isolate of each patient. Follow up isolates are linked to their phage profile only when: i. their CT differs from the CT of the isolate before, or ii. their phage profile differs from that of the isolate before.

The study’s collection comprises 197 longitudinal isolates from 12 CF patients infected by a *P. aeruginosa* CT for a continuous period of at least 4 years. These isolates were routinely sampled from patients seen monthly at the out-patient CF clinic at Rigshospitalet. Sputum samples were taken for culture, biobanking of *P. aeruginosa* isolates, and antibiotic susceptibility testing. The biobanked isolates were also sequenced and clone-typed as part of patient treatment while attending the Copenhagen CF Center at the University Hospital, Rigshospitalet, Denmark. To better explain the terms ‘CT’ and ‘isolate’, Fig. 1(a) can be used. There, a patient’s infection history (drawn as a horizontal line) is characterized by all the bacterial strains isolated from that patient, i.e. what is commonly termed as ‘bacterial isolates’ or simply ‘isolates’. These isolates can be classified into one or more CTs. As an example, in the infection history of patient PID08309 we have 14 isolates, which are represented by dots and crosses, and these isolates belong to four different CTs, namely DK12, DK25, DK33, and DK26. Within a given bacterial species (here *P. aeruginosa*), isolates are classified into CTs based on genomic differences. In the case of Rigshospitalet’s *P. aeruginosa* biobank and thus of this collection, CTs were demarcated based on >10 000 differential single-nucleotide polymorphisms after *de novo* assembly and alignment of isolate genome sequences (Marvig et al. 2015). In total, the 197 isolates of this study’s collection cluster into 25 different CT. Across their infection history, patients that were found to be colonized by isolates of different CT are termed ‘polyclonal’ patients, while patients consistently infected by a single CT are termed ‘monoclonal’ patients.

Figure 1(a) presents the pattern of this study’s isolate collection over time for each of the 12 patients in the collection, including CTs showing persistent presence (hereafter referred to as persistent CT; shown as blue isolate lineages), as well as transient CTs (shown via CT labels of grey isolates). A persistent CT is defined as the CT that is longitudinally retraced the longest and for a period of at least 4 years within a given patient and all remaining CTs correspond to transient CTs (i.e. CTs that are retraced for a period shorter than that of the persistent CT within that same patient). Based on these definitions, 12 of the 25 CTs from the study’s collection were categorized as persistent, and 13 of the 25 CTs as transient. The 12 patients were selected according to the following criteria: (i) all 12 selected patients had a complete record of their infection history available, (ii) their very first *P. aeruginosa* and regular subsequent *P. aeruginosa* isolates were stored in the Clinic’s biobank, (iii) *P. aeruginosa* was detected in follow-up samples over a period of at least 4 years. Most were previously published (Marvig et al. 2015), whereas 25 additional isolates are first presented here to extend the timespan of this collection. Detailed information about specific isolates such as patient of origin, year of collection, and prophage screening results are included in Table S1.

Illumina reads of the published isolates were extracted from https://www.ncbi.nlm.nih.gov/sra, cleaned with Cutadapt v4.4 (Martin 2011) and assembled with SPAdes v3.14.0 (Bankevich et al. 2012) choosing BayesHammer correction and careful mode. DNA from the new isolates was extracted with the DNEasy Blood and Tissue Kit (Qiagen) and libraries were built with Nextera® XT and sequenced on an Illumina MiSeq (250-bp paired end) or NextSeq (150-bp paired end).

Additionally, isolate PaLo43 was sampled from a CF patient attending the University Hospital of Leuven, Belgium, and strain PAO1 was purchased from the DSMZ collection. PaLo43 and PAO1 served as phage indicator strains. For all experiments, bacterial cultures were grown overnight in Lysogeny Broth (LB)-Lennox broth and agar (Sigma-Aldrich) at 37°C and 200 rpm shaking.

### 2.2. Prophage predictions and longitudinal frequency counting

To predict active prophage-like elements, the longitudinal isolates of each patient environment were scanned with Prophage Hunter’s server (Song et al. 2019), choosing both default and ‘skip similarity matching’ options. Results were parsed with an in-house python script as follows: elements from the same CF airway environment were merged, and ‘active’ and ‘ambiguous’ elements were extracted and grouped under their corresponding ‘closest phage’ hit. Next, each group was listed in descending order of longitudinal frequency. To confirm that elements under the same closest-phage group were closely related, we additionally BLASTn-compared them using default settings. The final curated results were used to count frequencies of the various prophage-like elements to determine those likely significant to host long-term persistence. Specifically, elements were considered significant when they were often encountered in the ‘persistent’ CT, i.e. the CT that was longitudinally retraced for at least 4 years. Pf1-like prophage elements were disregarded as these have already been extensively studied (Knezevic et al. 2015, Burgener et al. 2019). For subsequent experimentation, we selected one early isolate per CF airway niche (hereafter referred to as representative isolates), provided its genome harboured all longitudinally frequent prophage-like elements. These 12 representative isolates were resequenced with Oxford Nanopore for genome completion (see following section), Nanopore assemblies were rescanned with Prophage Hunter and PHASTER (Arndt et al. 2016) and results were compared. Presence of detected prophages across the 197 screened isolates is shown in Fig. 1(b) and (c) (and supplementary material referenced therein). In Fig. 1(b), green colour marks isolates positive for prophage and purple colour marks isolates negative for prophage. In Fig. 1(c), green (positive for prophage) isolates are labelled with the names of those longitudinally frequent prophage-like elements, which could be induced by this study (see later sections).

### 2.3. Prophage genome annotations

The genome of each longitudinally frequent prophage-like element was annotated to separate any likely intact prophages from other elements (e.g. pyocins). For that, we combined autoannotations with manual annotations. Autoannotations were conducted with RAST’s annotation server v2.0 (Aziz et al. 2008) using the RASTtk annotation scheme and GeneMark-Glimmer (Besemer and Borodovsky 2005, Delcher et al. 2007) as gene callers. At this stage, if no or only tail-related structural genes were predicted, the element was deemed to not be an intact prophage and was excluded from further analysis. Predictions for proteins were verified with Blastp (Altschul et al. 1990), HHpred (Gabler et al. 2020), and InterProScan v5.62–94.0 (Jones et al. 2014), for tRNAs with tRNAscan-SE v2.0 (Chan and Lowe 2019), and Aragorn v1.2.41 (Laslett and Canback 2004), and a function was assigned when at least two predictions agreed. Prophage genomes were also scanned for genes encoding antimicrobial resistance and virulence factors with CARD (threshold of 80% identity over 40% coverage) (Alcock et al. 2023) and PHIB-BLAST (PHI-Base v4.14; threshold of 50% identity over 50% coverage, e-value <10^−3^) (Urban et al. 2022), respectively. Putative repressors were identified by gene cluster comparisons with known *Pseudomonas* prophages using Clinker v0.0.27 (Gilchrist and Chooi 2021) and running PHMMER searches against the ‘Reference Proteomes’ database (Potter et al. 2018). Final annotation maps were designed with SnapGene (www.snapgene.com). Longitudinally nonfrequent prophage elements were annotated via our autoannotation method and deemed likely intact provided that the major capsid protein gene and other structural genes, both capsid-related (e.g. portal and head-to-tail joining proteins) and tail-related (e.g. tape measure and sheath proteins), were predicted.

### 2.4. Nanopore whole-genome sequencing and *de novo* assembly

As additional corroborations of predictions and to fully resolve the genomic architecture of the strains, whole-genome sequencing of the 12 chosen isolates was expanded using the Oxford Nanopore long-read technology. This was done to prevent overlooking a prophage due to scanning low contiguity and low completeness assemblies (Jain et al. 2018). High-molecular-weight gDNA was extracted from overnight cultures with Genomic-tip 100/G (Qiagen) following the Qiagen Genomic DNA Handbook and a published protocol (Alvarez-Arevalo et al. 2023). Before quality control, DNA extracts were mildly sheared by 20x passage through a 25-G needle to encourage homogenization. Libraries were prepared with the SQK-RBK004 kit (Oxford Nanopore Technologies) for rapid barcoding and sequenced on MinION R9.4.1 flow cells. High-accuracy basecalling was conducted with Guppy v4.2.2 (github.com/nanoporetech) by specifying ‘–min_score_mask 40’ to reduce the number of false positives. Reads under 1000 bp and scoring below Q10 were removed using SeqKit v0.13.2 (Shen et al. 2016). Retained reads were assembled with Flye v2.9 (Kolmogorov et al. 2019) and assemblies were polished through four runs of Racon v1.4.21 (Vaser et al. 2017) to remove random sequencing errors, then Medaka v1.2.0 (github.com/nanoporetech) and Homopolish v0.2.1 (Huang et al. 2021) to remove systematic Nanopore errors. Polished assembly accuracy and genome completeness were assessed using BUSCO v5.1.3 (Simão et al. 2015) and CheckM v1.0.18 (Parks et al. 2015) against the Pseudomonadales database. We evaluated coverage per 1000 bp intervals with DepthOfCoverage of GATK v4.1.6.0 (Van der Auwera and O’Connor 2020) and sequenced deeper any genomes with scores <20x. These deep sequencing and post-assembly processing steps were conducted to generate reference long-read assemblies with a final depth ≥65x for all genomes.

### 2.5. Prophage induction and DNA extractions

For induction experiments, overnight cultures of the 12 isolates were diluted to an optical density (OD) of 0.1 in 9 ml LB and incubated at 37°C until early exponential phase (hereafter t0), which corresponded to ODs of 0.2–0.3. At t0 we harvested 700 ul per diluted culture, after adding either 2.5 μg/ml mitomycin C (hereafter referred to as mitC) or ~0.5x the minimal inhibitory concentration of ciprofloxacin (hereafter referred to as cipro; Table S2). Samples were immediately centrifuged (17 000 × *g*, 5 min, 25°C) and supernatants were passed through 0.45 µm cutoff cellulose acetate syringe filters (LABSOLUTE®) and placed on ice until needed. Meanwhile, the diluted cultures were reincubated and sampled again after 30 min, 1 h, 2 h, 3 h, 4 h, and 19 h. Similarly, we sampled diluted cultures in the absence of antibiotics to check for self-induced prophages.

Aliquots of the filtered samples were tested for induced prophages via double agar overlay assays (Kropinski et al. 2009). Briefly the overlays were produced using 4 ml of LB broth supplemented with 0.4% w/v agarose (Fisher Scientific) and 0.1 ml overnight of either PAO1 or PaLo43. Each sample was serially diluted tenfold and 3 × 10 ul per dilution were spotted against the indicator lawn. Following 24-h incubations, the resulting plates were inspected for individual plaques or signs of cell lysis at the position of the spots. All positive samples were sorted out to repeat double agar overlays, except that 0.1 ml of tenfold dilutions were now blended with the overlay.

To capture all induced prophages, DNA extractions were performed for both the positive samples and the 19-h-filtered samples of those inductions that yielded no lysis. We extracted 2 × 100 ul per sample and otherwise followed a published protocol (Moineau et al. 1994), with few modifications. Briefly, samples were passed through 0.45-µm-cutoff ultrafiltration spin-columns (Millipore), then incubated with 10 U of DNase I at 37°C for 2 h and for the next steps volumes were doubled. The two sample copies were loaded to the same purification column before the wash step and eluted with 25 ul of TE buffer (10 mM Tris–HCl, 0.1 mM EDTA, pH 7.5). Samples sequenced to contain high levels of bacterial-DNA-read ‘noise’ were re-extracted after pretreatment with 0.1 volumes chloroform, according to PoT protocol (Bonilla et al. 2016).

### 2.6. Induced prophage DNA sequencing and read mapping

The gDNA libraries of all induced prophage DNA samples were prepared with the Nextera Flex kit (Illumina) and sequenced with the Illumina MiniSeq using a paired-end approach (2 × 150 bp). Next, reads were quality-controlled with FastQC (Wingett and Andrews 2018) and scanned with Trimmomatic (Bolger et al. 2014) to remove adapter sequences, filter by length (>50 bp), and trim lower-quality regions (options: ILLUMINACLIP:NexteraPE-PE.fa:2:30:10LEADING:3 TRAILING:3 SLIDINGWINDOW:4:15 MINLEN:50). Reads were mapped onto the corresponding host genomes using bwa mem mapper (Li 2013) with default options and resulting mapping files were visualized with WeeSAM (https://github.com/centre-for-virus-research/weeSAM) and UGENE v42.0 (Okonechnikov et al. 2012). Genomic regions corresponding to induced prophages were hereby localized with high border accuracy, and a genomic region was categorized as an induced prophage region when its depth of coverage was >20x (see corresponding maps attached in Fig. S1). Sole exception was prophage Bise, where three mapping results had to be combined to resolve this prophage’s genome. The graph of induced versus uninduced prophages per isolate was generated with GraphPad Prism v.10.0.0.

### 2.7. Comparative genomics, phylogenetics, and CRISPR-Cas system predictions

The reference long-read genome assemblies of the 12 isolates were aligned using Parsnp version 1.5 (Treangen et al. 2014) and a tree was generated with RAxML (Stamatakis 2014) on single nucleotide polymorphisms (SNPs) identified from the alignment. The tree was annotated with support values from 500 bootstraps and visualized with iTOL v6.7 (Letunic and Bork 2007). CRISPR-Cas systems of the 12 isolates were predicted and classified with CRISPRCasTyper v1.8.0 (Russel et al. 2020) using ‘Circular topology’ and, otherwise, default settings. Self-targeting against own intact prophages was investigated by first extracting the list of spacers predicted per isolate genome and adding the protospacer adjacent motif (5'-GG-3' for I-F predictions and 5'-CAT-3' for I-E predictions) upstream of each spacer sequence. Secondly, the edited spacers were aligned against their own prophage genomes using the Megablast algorithm.

Prophage pairwise intergenomic similarities were computed via web-based tools VIRIDIC (Moraru et al. 2020) and ViPTree (Nishimura et al. 2017) using default settings. For that, all prophages deemed intact were compared amongst them and against a custom, literature-based database of all *P. aeruginosa* prophages proven to exist as active particles (Pubmed search ‘Pseudomonas’ AND ‘lysogenic’/’prophage’/’excis?’/’temperate’; Table S3). Having sequenced the genome of all induced prophages, we verified their presence in longitudinal isolates using the BLASTn algorithm and extracted their genome sequence from the latest isolate they lysogenized with CLC Genomic Workbench V8.0 (Qiagen). Using EasyFig v.2.2.5 (Sullivan et al. 2011), extracted sequences were linearly BLASTn-compared to the corresponding sequenced prophage genome. The phylogenetic tree based on concatenated amino acid sequences of repressor/antirepressor was constructed via NGPhylogeny.fr (Lemoine et al. 2019) with ‘PhyML + SMS/OneClick’ for the tree inference.

## Results and discussion

### 3.1. Patterns of induction of 15 identified, intact prophages

Our bioinformatics analysis identified a sum of 29 intact prophages in the genomes of the 197-isolate collection (Table S1 and Fig. 1(a) for the collection). These prophages were also found in the genome of the 12 representative *P. aeruginosa* clinical isolates that make up part of the 197-isolate collection (Fig. 1(b) and (c)). Prophage Hunter generally outperformed PHASTER in predicting prophage completeness (Table S4). Double agar overlay assays were the first tests undertaken to identify free phages resulting from our induction experiments. However, these assays alone would have been insufficient to distinguish whether lawn lysis stems from phage rather than bacteriocin killing. Such level of detail was possible thanks to the sequencing analysis. By mapping lysate reads to their corresponding host genome we could identify induced prophage locations with high border accuracy.

**Figure 2.**
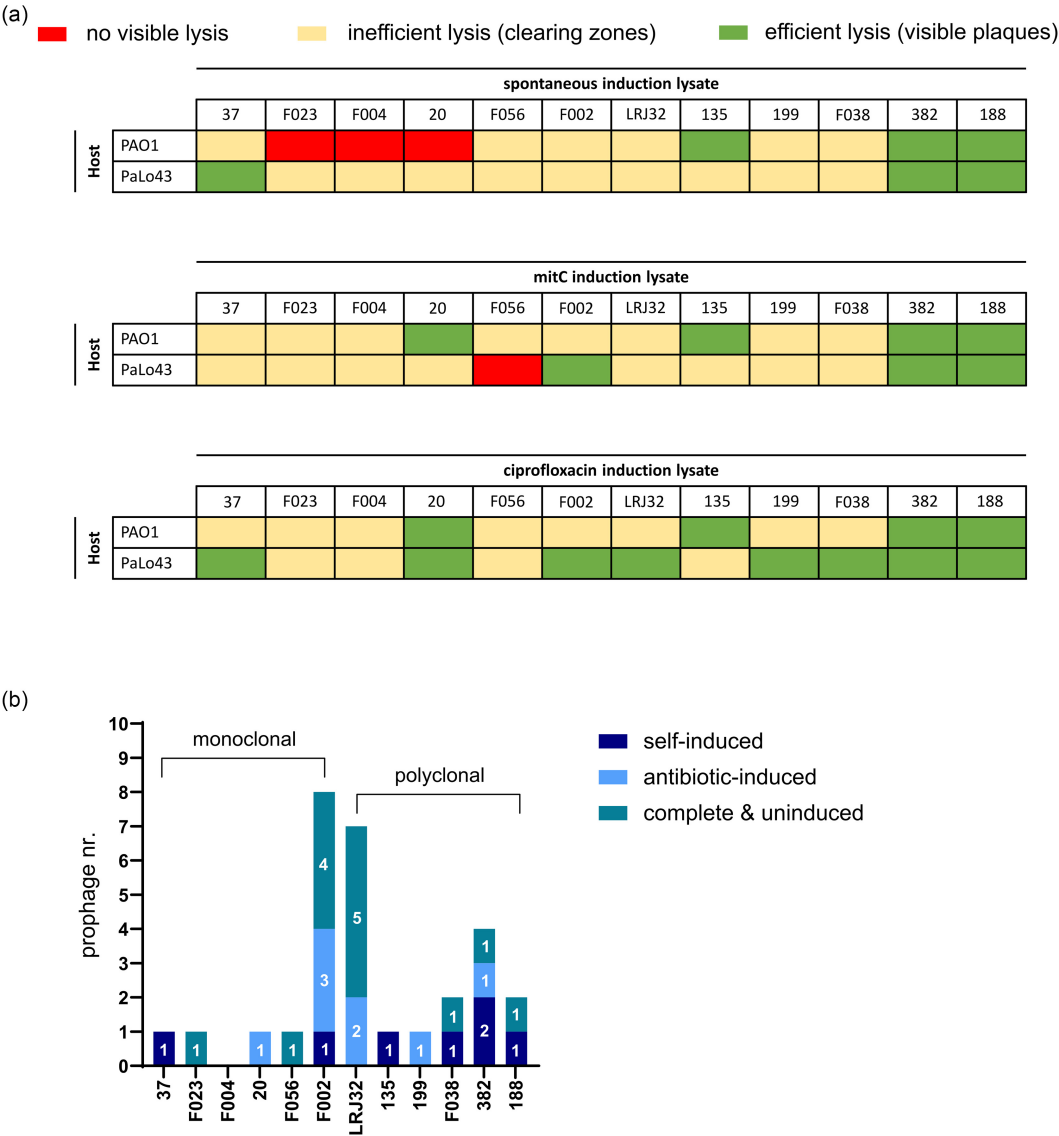
(a) Overview of the double agar overlay outcomes. The supernatants of induced or uninduced overnight cultures were plated against the lawns of PAO1 and PaLo43. Visible lysis, i.e. lysis where individual plaques were counted, is marked in green, clearing zones are judged as inefficient lysis and marked in yellow, and lysis absence is marked in red. Prophage inductions were mostly correctly implied or indicated via the noted clearing zones and plaques, as confirmed by the quantitative sequencing results; (b) Quantitative results of induced prophages via mapping of lysate reads to gapless host genome assemblies. Host strains (referred to as ‘isolates’ in main text) are divided into two groups—those isolated from patients with apparent monoclonal infections (i.e. infections by a single *P. aeruginosa* CT; see main text for definition of CT) and those with polyclonal infections (i.e. infections by multiple *P. aeruginosa* CTs). Different shades of blue indicate whether a given prophage was only induced by an antibiotic or could also self-induce and a teal colour denotes that a prophage was predicted as intact but not induced with the methods tested. The CTs of the presented strains are: strain 37–CT DK04, strain F023–CT DK45, strain F004–CT DK17, strain 20–CT DK07, strain F056–CT DK06, strain F002–CT DK12, strain LRJ32–CT DK18, strain 135–CT DK15, strain 199–CT DK29, strain F038–CT DK31, strain 382–CT DK26, and strain 188–CT DK26.

In total, 15 predicted-as-intact prophages were found to exist as free particles by lysate sequencing (Fig. S1) with prophage Bise being variably induced with mitC and Sirocco being variably self-induced. The isolates harbouring one or more of these 15 prophages are presented in green font in Fig. 1(b). The specific prophages among these 15 that are present in each of the green isolates are labelled in Fig. 1(c) and listed in Table S1. While we did always predict genes encoding a major capsid protein along with other structural capsid- and tail-related proteins for the remaining 14 prophages (see Table S5 for Zenodo link to genome and annotation files), no or scarce reads corresponding to them were captured (Fig. 2(b) and Fig. S1). Given that lysate DNA was extracted from at least two different time points per isolate and type of induction, it is unlikely that our method missed induced dsDNA prophages. Explanations for why the remaining 14 prophages were not induced can be attributed to various factors. For example, lysogenic to lytic conversion for these prophages may be triggered by conditions different to the ones tested (Nanda et al. 2015), or they may belong to a noninducible class of temperate phages (such as Escherichia virus P2) (Bertani 1951). Domestication is also likely to have rendered some of the 14 prophages defective either via extended gene loss that reduced genomes to <30 kb or via more limited and targeted gene loss that converted them to capsid- and tail-encoding particles known as killer particles and gene transfer agents (Bobay et al. 2014). A last improbable scenario is that, while these prophages excised, our DNA filtration and Illumina sequencing methods selected against them because of their ssDNA genome (Kleiner et al. 2015).

Looking back at the results of the double agar overlay assays (Fig. 2a), we noted that clearing zones and plaques did always result from prophage induction, as confirmed from the sequencing (Fig. S1 and Fig. 2b). Of the induced prophages, some self-induced while others excised due to one or both antibiotics used (mitC or cipro; Fig. 2b). Under the tested *in vitro* conditions, self-induction was common and occurred for almost 50% of the prophages sequenced as induced. This aligns with multiple studies on *P. aeruginosa* clinical isolates from CF patients, such as (Miller and Rubero 1984, Essoh et al. 2013), which reported frequent *in vitro* prophage self-induction. Cipro and mitC caused additional prophages to excise from the 12 isolates (Fig. 2b). In particular, cipro induction patterns corroborated and occasionally expanded those of mitC (Fig. 2a). Ciprofloxacin, a fluoroquinolone commonly prescribed to CF patients including the Copenhagen CF Center patients, was previously shown to trigger high rates of *in vitro* prophage induction in clinical *P. aeruginosa* (Fothergill et al. 2011).

Considering these *in vitro* observations, we can extrapolate that *in vivo P. aeruginosa*-infected CF airways often contain high titres of excised prophages, as already showcased for isolate LESB58’s prophages (James et al. 2015). This extrapolation can be further supported by the regularity of prophages in genomes of *P. aeruginosa* CF airway isolates. Our results support findings in other studies that most clinical *P. aeruginosa* are mono- or polylysogenic (Miller and Rubero 1984, Essoh et al. 2013), because except F004 all isolates harboured prophages, with 5 of 12 being polylysogens harbouring two to eight intact prophages (Fig. 2b). It is tempting to hypothesize that in the CF airways environment, lysogeny (especially polylysogeny) contributes to *P. aeruginosa* persisting longer than its nonlysogenic or prophage-poor counterparts, as already shown for the wound environment (Marshall et al. 2021). This could be due to prophage integration disrupting relevant host genes, but also due to moron genes and/or superinfection exclusion. Intriguingly, in our study monolysogeny was indeed almost exclusively associated to isolates from monoclonal infections, except for isolate F002 (Fig. 2b). Yet, the CT of F002 (DK12) was reported to occur in multiple patients (Bartell et al. 2021) likely implying its direct acquisition from a polyclonal CF airway environment.

To explore possible correlations between polylysogeny of an isolate and a higher permissiveness to phage invasion, we performed *in silico* predictions of CRISPR-Cas systems. In line with other studies (Wheatley and MacLean 2021), our study revealed that isolate genomes containing functional CRISPR-Cas systems harboured one or zero prophages (Table 1). Despite the reported strong association between type I-F systems and self-targeting (Nobrega et al. 2020, Wheatley and MacLean 2021), we detected no spacers targeting those native intact prophages. Conclusive answers to the above would necessitate experimental validation of the predicted CRISPR-Cas systems, and of any phage countermeasures.

**Table 1.** List of studied isolates predicted to contain functional or orphan CRISPR-Cas systems as compared to the number of intact prophages harboured by each isolate.

Isolate	Number of Cas operons	Predicted Cas subtypes	Number of associated CRISPR arrays	Number of intact prophages	Native prophage (self-) targeting?
37	1	I-F	2	1	No
135	1	I-E	2	1	No
199	1	I-F	2	1	No
F004	1	I-F	2	0	No
F023	1	I-F	2	1	No
F056	1	I-F	2	1	No
LRJ32	–	Orphan	1	7	–

### 3.2. Identified intact prophages show high genomic diversity

For the rest of this study, we focus mainly on 15 of the 29 intact prophage genomes, which could be resolved with high-border accuracy because we could induce them experimentally. We present their names and key genomic characteristics in Table 2, which elucidates the diverse %GC content within this prophage collection as well as mixed presence and arrangement similarity of tRNAs. Of the prophage genomes found to encode tRNAs (Table 2), Marin and Haboob displayed an almost identical tRNA gene arrangement, despite their overall nucleotide dissimilarity (94% identity over 46% query cover by BLASTn). Importantly, these same genomes had the highest %GC content disparity as compared to *P. aeruginosa*’s genome, which averages 66.6% (Hesse et al. 2018). It is hypothesized that phages carry selected tRNA genes either to compensate for any codon usage differences with their host or to overcome host tRNA-depleting antiphage strategies (Bailly-Bechet et al. 2007, Hesse et al. 2018, van den Berg et al. 2023). Our observation regarding the %GC content disparity would be better explained by the former theory. Collectively considered, tRNA presence and %GC content disparity could imply that the implicated prophages encountered various hosts throughout their evolutionary history as temperate phages and may explain how these prophages evaded host domestication and remained inducible.

**Table 2.** Names and key genomic characteristics of the induced *P. aeruginosa* prophages. The ends of all genomes were defined based on their integration in the host chromosome.

Prophage	Genome size (bp)	Lysogenized isolate	ORFs with assigned function	%GC content	tRNA genes	Repressor, antirepressor
Rashabar	38 722	37	16/55	63.3	–	unclear, Ner-like
Riah	44 821	20	24/59	62.1	–	CI-like, Cro-like
Alize	38 819	F002	27/65	61.9	–	CI-like, Cro-like
Haboob	50 033	382	26/85	59.7	tRNA^Met^ tRNA^Gly^ tRNA^Asn^ tRNA^Thr^	CI-like, Cro-like
Marin	51 941	F002	29/84	59.9	tRNA^Gly^ tRNA^Asn^ tRNA^Thr^	CI-like, Cro-like
Shamal	44 124	F002	19/76	57.3	tRNA^Ser^	CI-like, Cro-like
Gregale	36 566	F002	15/53	64.2	–	CI-like, Ner-like
Solano	40 334	LRJ32	24/62	62	–	CI-like, Cro-like
Meltemi	36 609	LRJ32	15/54	64.5	–	CI-like, Ner-like
Lodos	44 613	135	24/55	63.9	–	CI-like, unclear
Bise	38 458	199	30/55	66	–	CI-like, Ner-like
Turba	42 092	F038	22/51	63.8	–	CI-like, unclear
Ostro	48 846	382	27/70	62.4	–	CI-like, Cro-like
Etesian	37 990	382	20/58	64.3	–	CI-like, Ner-like
Sirocco	36 945	188	35/52	62.5	–	CI-like, Cox-like

Seeking fitness- and persistence-relevant features, we performed functional gene annotations with a special focus on moron-encoding genes. Moron proteins (aka morons) are recognized as important contributors to the fitness of *P. aeruginosa* and other bacteria through, for instance, antimicrobial resistance or virulence regulation (Tsao et al. 2018). Depending on their functionality and genomic background role, some could hence support persistence of the prophage host in the CF airways. In Table 3, we show the six morons we found distributed across five of our prophage genomes. Outside moron genes, functions could on average be assigned to one-third of the predicted open reading frames (ORFs; Table 2); annotated ORFs were typically associated with morphogenesis, DNA packaging, and lysogeny–lysis modules due to these proteins being more conserved in phages. Transposition was predicted for prophages Bise, Etesian, Gregale, Meltemi, Marin, and Rashabar and agrees with early reports on the wide distribution of transposable phages in *P. aeruginosa* (Akhverdian et al. 1984). The ability to transpose in the host genome means that such prophages generate various mutations available to selection, assisting the host with adapting quickly to the challenging niche of the CF airways and the varying antibiotic treatments. This has been established for LESφ4 (Davies et al. 2016b, O’Brien et al. 2019). Their ability to transpose may hence explain why retaining the inducibility of such prophages would be favoured by their host.

**Table 3.** List of all moron proteins predicted to be expressed by the genomes of intact and inducible prophages identified by this study. Bcr was predicted by screening against the CARD database. All other proteins were predicted using PHI-Base.

Predicted morons	Prophage	Encoding gene (% identity)	Function
Bcr (NCBI: ALV80601.1)	Ostro	peg. 66 (92.5%)	Grants resistants to bicyclomycin by selectively inhibiting Rho (Kohn and Widger 2005)
CcoN4 (PHI:7765)	Ostro	peg. 69 (100%)	Promotes pathogenicity and biofilm growth for strain PA14 in a *Caenorhabditis elegans* infection model (Jo et al. 2017)
YdcR (PHI:7225)	Ostro	peg. 70 (55%)	Promotes pathogenicity of *Salmonella enterica* in a murine infection model and directly regulates virulence factor SrfN (Liu et al. 2017)
AmrZ (PHI:5372)	Solano Alize	peg. 55 (50%) peg. 10 (50%)	Represses cellulose biosynthesis and likely regulates several virulence factors of *P. syringae* (Prada-Ramírez et al. 2016)
AlgR (PHI:8158)	Haboob	peg. 67 (34%)	Activates the biosynthetic pathway of alginate, which leads to mucoidity, immunoevasion, and eventually persistence of *P. aeruginosa* in CF (Pedersen et al. 1992)
			Contributes to virulence via the regulation of type IV pili, which are essential for *P. aeruginosa* mammalian cell colonization, competence, and pathogenesis (Burrows 2012)
DprA (PHI:11059)	Riah	peg. 58 (43%)	Its deficiency leads to *Streptococcus pneumoniae*’s attenuated virulence in a bacteremia mouse model infection (Lin and Lau 2019)

**Figure 3.**
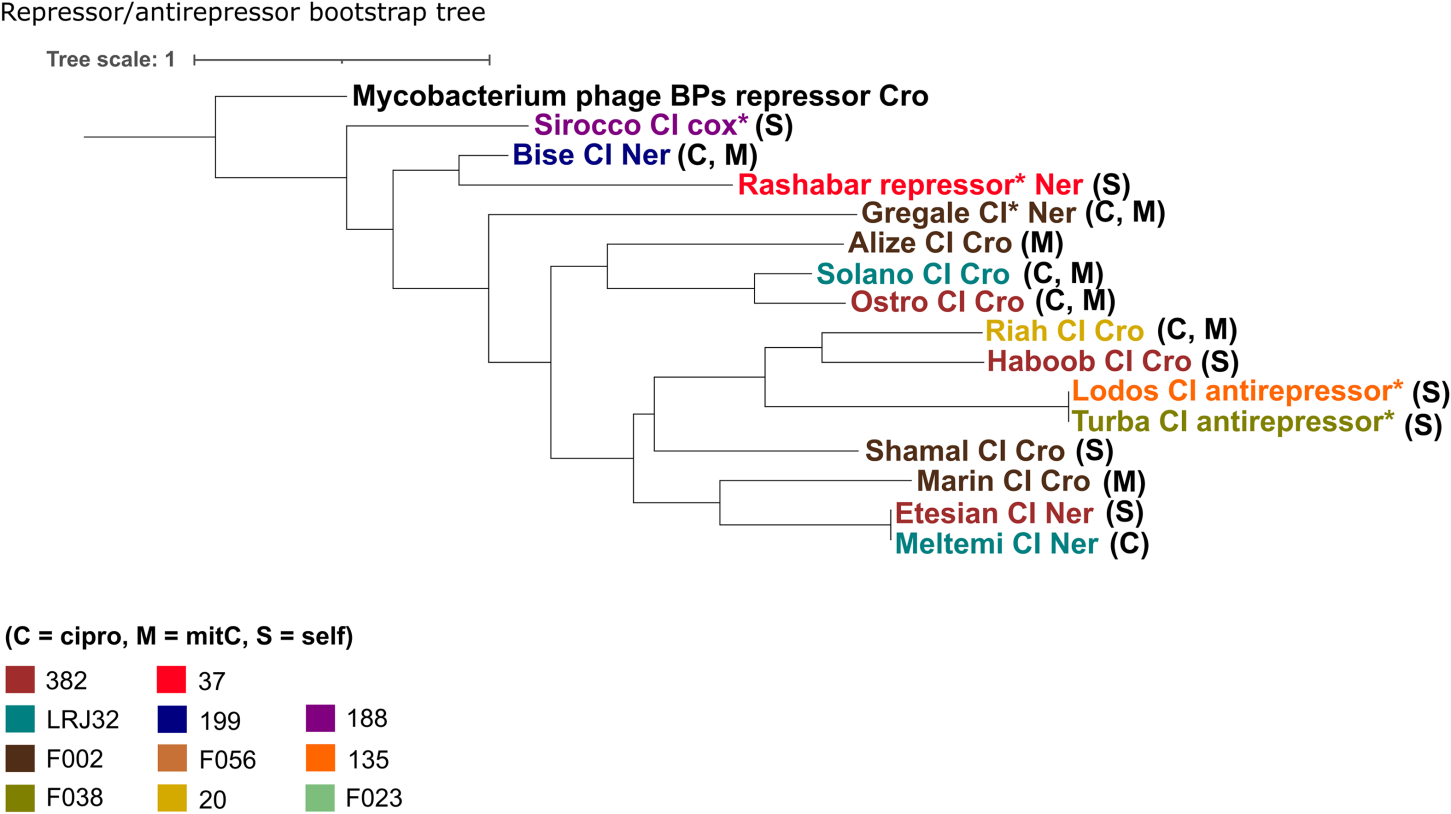
Bootstrap tree of predicted amino acid sequences of the concatenated repressor/antirepressor built using NGPhylogeny.fr and the ‘PhyML + SMS/OneClick’ workflow. Branch tags are coloured according to prophage. Prophage induction profiles are noted to the right of a tree branch as cipro (C), mitC (M), or self-induced (S).

Other noteworthy annotations were one unclassified toxin–antitoxic system (TA) encoded by Shamal’s genome, one death-on-curing (Doc) toxin family protein encoded by Sirocco’s genome and one pyocin activator PrtN family protein encoded by Lodo’s genome. TA systems are encoded by prokaryotic genomes and can be involved in regulating biofilm formation, motility, and antibiotic resistance (Shmidov et al. 2022). They can also function as prokaryotic antiphage defence mechanisms that trigger abortive infection, where infected cells commit suicide to prevent the spread of phages within the population. While TAs are widespread in prokaryotic genomes, the adoption of TA systems by phages is indeed not unheard of. For instance, *P. aeruginosa* phage Pf4 genome harbours a TA that controls production and superinfection exclusion of Pf4 in PAO1 (Schmidt et al. 2022). The toxin Doc is part of a TA system called P1 plasmid addiction operon and is neutralized by its cognate antitoxin (prevent-host-death; PhD) unless if the prophage is lost. In that occasion, PhD is proteolyzed resulting in Doc release and cell death (Lehnherr et al. 1993, Smith and Magnuson 2004). Thus, the presence of TAs in the genomes of Sirocco and Shamal is likely to confer a selective advantage to these prophages, because their TAs are expected to secure stable maintenance of the prophages within the host population and contribute to host survival and adaptation. Similarly, expression of a pyocin activator protein by prophage Lodos could activate a pyocin-type bacteriocin (Matsui et al. 1993) predicted to be located upstream of Lodos (pyocin: 4 177 288–4 221 906 bp, Lodos: 4 779 268–4 799 021 bp) in the bacterial chromosome of its host isolate (135). This can likely enable 135 and same-CT successor isolates to better compete and prevail against other bacteria in their polyclonal CF airway niche (Michel-Briand and Baysse 2002). To investigate induction patterns of the 15 discussed prophages, we combined functional annotations and synteny maps to related phages, which suggested that the lysis–lysogeny switch was regulated by repressor CI and either Cro- or Ner-like antirepressors for most prophages (Table 3). However, a combined repressor/antirepressor tree (Fig. 3) failed to cluster prophages according to their induction pattern, suggesting that repressor/antirepressor genes are not alone in governing lysogeny–lysis decisions.

To explore how diverse the 15 induced and the 14 uninduced prophages were as compared to an external—yet related—prophage population, we built a database of previously published dsDNA genomes belonging to 26 *P. aeruginosa prophages* proven to exist as active particles (Table S3). Intergenomic similarity scoring for all 55 genomes was performed with VIRIDIC with default settings that follow the thresholds for species (<95%) and genus (∼70%) demarcation set by the International Committee on Taxonomy of Viruses (Moraru et al. 2020). Overall, this analysis produced genera clusters made of the following database versus novel prophages: phiCTX-Dobby with Harmattan (uninduced) and Sirocco (variably induced), JBD5-D3112-MP29 with Etesian (induced), JBD30-pfII40a with Gregale (induced) and Meltemi (induced), H71-H72-JBD25-Ps56 with Caju (uninduced) and Notus (uninduced), and JBD67 with Rashabar (induced) (Fig. S2). We then evaluated whether any features of relevance to infection and persistence found in database prophages were also present in our prophages. JBD5 possesses two genes encoding anti-CRISPR (Acr) proteins, AcrIF3 (NCBI:YP_007392740.1) and AcrIE1 (NCBI: YP_007392738.1), which are encoded by genes peg.25–6 in Etesian. These proteins should enable Etesian’s spread to other *P. aeruginosa* that, unlike studied host isolate 382 (DK26), carry active type I-F and I-E CRISPR-Cas systems (Trasanidou et al. 2019). A wider host range suggests that Etesian likely assisted DK26 to prevail over competing CTs in its polyclonal CF airway niche. While we did not find the cytotoxin that is present in phiCTX (NCBI: NP_490598.1) within our two clustered prophages, we did note that phiCTX is a member of the P2 noninducible prophage class. Thus, the grouping of prophages Sirocco and Harmattan with phiCTX is consistent with our experimental difficulties inducing them. Like with phiCTX, eventual lysis may occur by mutations in a lysogeny-associated gene (Nakayama et al. 1999). For even broader comparisons, this study’s prophage genomes were BLASTn-compared to the viral nucleotide collection database (Table S6).

**Figure 4.**
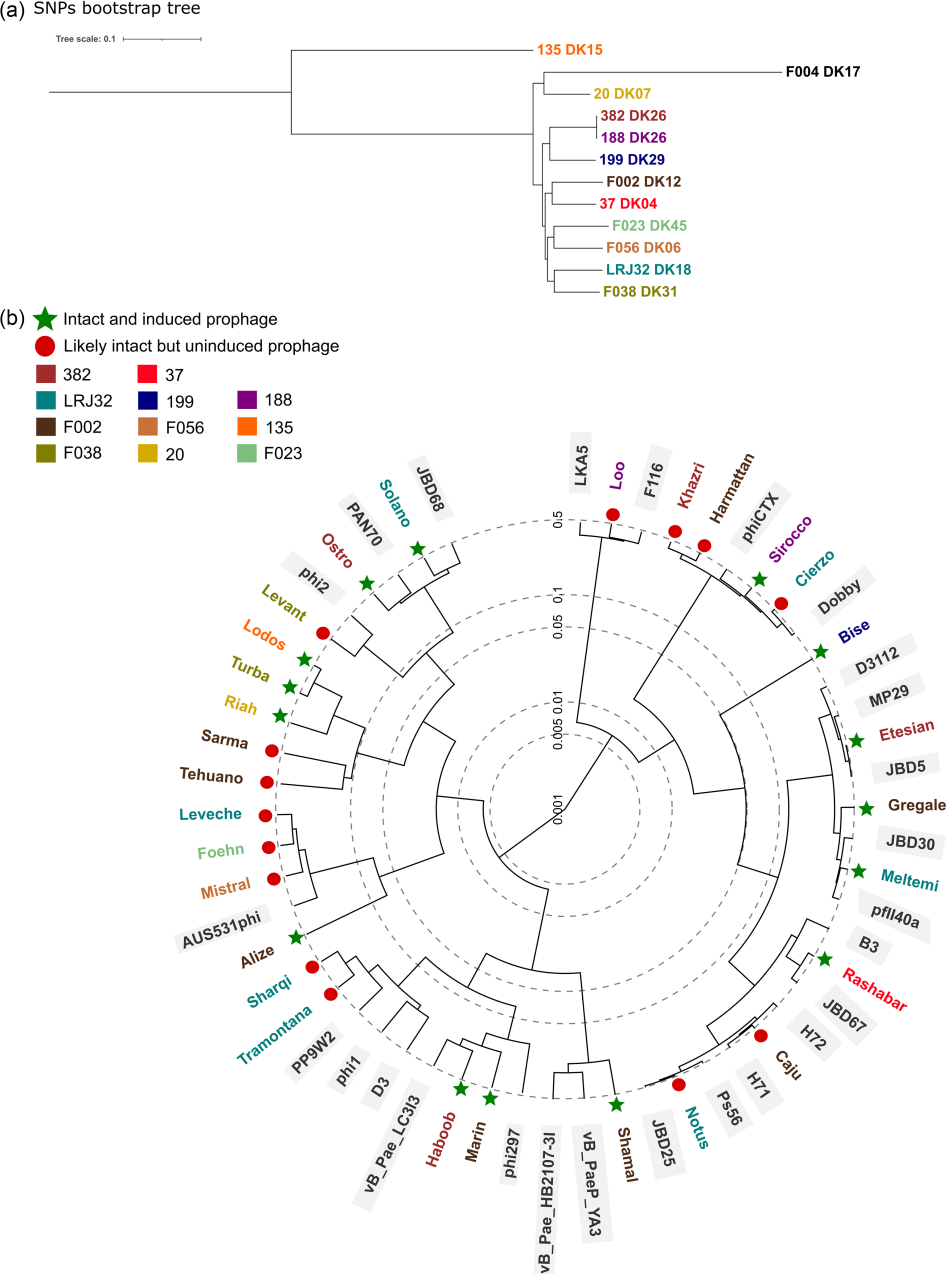
(a) Bootstrap tree based on SNPs among the genomes of the 12 studied isolates (please find definition of term ‘isolate’ in main text), and (b) proteomic tree computed with tBLASTx in ViPTree shows the clustering of study’s prophages versus related literature prophages of *P. aeruginosa* shown to exist as particles (also referred to as ‘database prophages’ in text). Colours follow the scheme of Fig. 3. Intact and induced prophages are signified by a green star and likely intact but uninduced prophages by a red circle. Database prophages are highlighted using grey background.

To further assess the level of diversity among these prophages, we compared results yielded by VIRIDIC with a ViPTree-built phylogeny. ViPtree constructs trees based on tBlastX similarities, hence increasing resolution of genomic relations. The derived tree (Fig. 4b) validated VIRIDIC and BLASTn results, highlighting the clade of active prophages Lodos–Turba–Riah and the monotypic taxon Bise for their very low similarity to any known prophages, which we interpret as novel prophages discovered via this study. Other active prophages with reduced similarity to known prophages were Haboob, Marin, Shamal, Alize, Ostro, and Solano. Phylogenetically related prophages (Fig. 4b) were not restricted to phylogenetically related hosts (Fig. 4a), which combined with the 29 prophages’ shared ecological niche, corroborates prior findings on the remarkable versatility and diversity of *P. aeruginosa* prophages (Johnson et al. 2022).

### 3.3. The genomes of longitudinally persistent prophages avoid gene loss and degradation over long evolutionary times

To assess the potential fitness contributions of the novel and diverse prophages we have been discussing, we return to their original evolutionary context. Each of the 12 *P. aeruginosa* isolates that harbour the 29 intact prophages was collected from a unique CF patient persistently infected by *P. aeruginosa*. To gather more evidence on whether any of these prophages favours host fitness and persistence, we gauged how frequently they appear longitudinally. Per patient environment, sequenced longitudinal isolates spanned a period of at least 4 years (Fig. 1). Here, we focus on the 15 intact and induced prophages, as complete genomes of the 14 uninduced prophages could not be verified with lysate sequencing. All same-patient longitudinal isolates were BLASTn-compared to the complete genomic sequence of each induced prophage to confirm earlier bioinformatics-determined frequencies. This approach was taken, because sole reliance upon low-contiguity Illumina-based assemblies for prophage discovery could lead to false-negative low prophage frequencies.

We found all 15 induced prophages to be present in at least 80% of the dates when a persistent CT was isolated, including the most recent date (Fig. 1b and c and Table S1). Hence, all 15 induced prophages were deemed longitudinally frequent, or else persistent. We present this information in the context of the CT background and persistence in patients across the entire isolate collection in Fig. 1(b), using green to highlight the isolates where longitudinally persistent and induced prophages were detected. From Fig. 1(b), we can see that more isolates from the persistent CTs carry longitudinally persistent and induced prophages, while isolates from transient CTs do not carry such prophages. To investigate whether such prophages are associated with persistent CTs, we evaluated their presence within clonal lineages, which are defined by the pairing of CT and patient background. As an example, in patient PID08309, we have three transient lineages (PID08309-DK12, PID08309-DK25, and PID08309-DK33), while we have one persistent lineage (PID08309-DK26). However, three persistent lineages were excluded from this investigation because we could not categorize them as positive or negative to longitudinally persistent and induced prophages. Specifically, lineages PID00037-DK06 and PID20509-DK45 were positive to longitudinally persistent prophages, which the authors could not induce, and lineage PID16905-DK17 was negative to longitudinally persistent prophages. In total, 2 of 15 transient lineages carry longitudinally persistent and induced prophages, while 9 of 9 persistent lineages carry such prophages, and a Fisher’s exact test indicates a highly significant association between inducible phage and persistence of lineage (*P* < .0001). Notably, this approach has important caveats related to lineage independence and repeated sampling over time. With respect to independence, we include two lineages associated with CT DK26, as DK26 persists independently in two patients, PID08309 and PID42824. We do not have genetic evidence of a direct transmission between these patients, and each CT survives in an independent environment under different treatment conditions and different competing CT, but the DK26 lineages are not entirely independent due to shared genetic background. With respect to repeated sampling, we evaluate the presence of lineages rather than isolates to reduce the bias of sampling over time, where the number of isolates from persistent CTs available for testing is inherently greater than the number of isolates available from transient CTs. However, the number of isolates collected from persistent lineages is still inherently greater than those collected for transient lineages, and prophages are not detected in all isolates from these persistent lineages. In a count of isolates instead of lineages, the latter phenotype matches that of isolates from transient CTs. A generalized linear mixed model would be a more robust starting point to modelling this repeat sampling issue, but the current study does not include a sufficient number of lineages to fit such a model, and we leave this for future work with an extended isolate collection. In summary, the statistical significance of the association between inducible prophage carriage and persistence of CT is a promising finding deserving of follow-up investigations.

**Figure 5.**
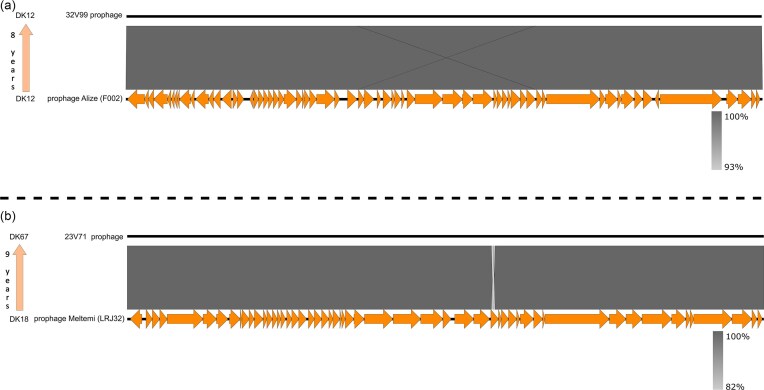
BLASTn similarity genomic synteny maps designed with Easyfig. A sequenced prophage from an early isolate is compared to its own genome identified in a later longitudinal isolate of the persistent CT (a definition of the terms CT and isolate is presented in main text), with the term ‘persistent’ used here to describe the CT that was longitudinally retraced the longest and for a period of at least 4 years within that patient (see also Table S5). (a) The genomic organization of prophage Alize, induced from isolate F002, is compared to that of a prophage harboured by a longitudinal isolate of the same CT (DK12) 8 years later. (b) The genomic organization of prophage Meltemi is compared to a prophage harboured by a longitudinal isolate belonging to the persistent CT (DK67) 9 years later. Meltemi is originally induced from isolate LRJ32 of a transient CT (i.e. a CT that is retraced for a period shorter than that of the persistent CT) but, as for all intact prophages harboured by LRJ32, it is later retraced in the persistent CT (DK67).

Turning to the genomes of the induced prophages, we observed minimal genomic alterations in all cases and throughout the extensive evolutionary time frame of 4–9 years that we considered. Figure 5 displays two examples of this genomic conservation; a monoclonal infection exemplified by the synteny map of prophage Alize and a polyclonal infection exemplified by that of Meltemi. The genome of Alize matched by 93% overall nucleotide similarity a region detected 8 years later in isolate 32V99 of the same CT (Fig. 5a). Similarly, the genome of Meltemi matched by 82% overall nucleotide similarity a region detected 9 years later in isolate 23V71 of the persistent CT DK67 (Fig. 5b). Additional synteny maps are available in Fig. S3.

Meltemi and its cohabiting prophage Solano constituted the only cases of a persistent prophage likely moving from a transient (DK18) to the persistent CT (DK67). We wanted to validate this hypothesis, versus the possibility that Meltemi and Solano were inherent features of DK67’s backbone. Indeed, the two phages were not found in 36V42, an isolate of DK67 which only transiently infected a patient. We extended this strategy and searched for additional CTs that persisted in one patient but transiently infected another and found examples for DK12 and DK26. DK12 persisted in patient PID76609 and was transient for patient PID08309 (Fig. 1). We screened the genomes of isolates F042, F043 and 93 from transient DK12 against the genomes of persistent prophages Alize, Marin, Shamal, and Gregale of isolate F002 (DK12). Again, we found no homologous sequences, which indicated that these prophages do not belong to DK12’s backbone. Another CT, DK26, was persistent in patients PID42824 and PID08309, which were represented by isolates 382 and 188. We found none of the four induced and two uninduced prophages from these isolates to be shared except in one case; Sirocco had 76% overall nucleotide similarity to a region in the genome of 382. The latter corresponded to the predicted as intact but uninduced prophage Khazri (Fig. 4b), which was found to be a longitudinally frequent element of 382 (Table S5). To further clarify the case of Sirocco, we looked at the genomes of isolates F011, F044, and 24V78. F011—sampled in 2002—and 24V78—sampled in 2021—originated from the same CF patient and a region of 99% overall nucleotide similarity to Sirocco was traced in both. The infection by DK26 in this patient seemed to prevail for almost 19 years, but we did not have sequences of intermediate isolates to validate its persistence. However, F044 from a CF patient transiently infected by DK26, also harboured a region with 99% overall nucleotide similarity to Sirocco. This identified ubiquity of Sirocco-type prophages in DK26 isolates combined with the general noninducibility of this prophage lineage (Fig. 4b) suggests that Sirocco (and Khazri) is an inherent part of DK26’s backbone.

### 3.4. Evolutionary context of prophages within *P. aeruginosa* persistence in the CF airways

Active prophages are bacterial parasites that burden the host in a dual manner; they are metabolically costly and can revert to the lytic cycle resulting in host death. Thus, the trend would be that they undergo mutational degradation along with other parts of the bacterial genome and become ‘grounded’ over time (Khan et al. 2020), unless they confer ecological and evolutionary benefits to their host (Harrison and Brockhurst 2017). In our study, we do not observe substantial degradation of prophage genomes or gene loss. Conversely, a prophage carrying a moron gene that provides fitness benefits unmatched by the host genome is predicted to persist within the host, even amidst conflicting selection pressures (Bailey et al. 2023). In our study, we detected persistence of prophages alongside the persistence of their host in the CF airways, and 7 out of 17 of these prophages could produce proteins with similarity to known morons (i.e. fitness-relevant functions). Six of the remaining prophages could, provided that inducibility is maintained, transpose in the host chromosome thus accelerating host evolutionary adaptation. Stability in the host environment is estimated to further prolong maintenance of such prophages (Bailey et al. 2023). Indeed, stability is a trait that characterizes *Pseudomonas*-dominated CF airways (Hampton et al. 2021), and likely creates a positive feedback loop between beneficial prophage maintenance and host persistence. Thus, we suspect that, excluding Sirocco, all other induced prophages likely enhance their hosts’ fitness and persistence in the CF airways. The ease of induction for nearly all prophages (except Sirocco and Bise) can, besides the already discussed advantages of transposition and competitor exclusion, ultimately enable beneficial transmission events where morons can be carried between different hosts and new morons can possibly even be introduced back to the origin population (autotransduction) (Haaber et al. 2016). However, ease of induction is also obvious evidence of prophage selfishness and opportunism in that mobility is preserved and replication in other CTs is possible. We propose that reported behaviours of intact prophages, whether as selfish genetic elements pursuing their own survival and proliferation or as nearly domesticated elements contributing to host adaptation and fitness, should not be regarded as mutually exclusive. Instead, they should be recognized as interconnected aspects of the nature of intact prophages, used to aid their survival. This idea is supported by Quistad et al. (2020), who reason that genetic elements (e.g. intact prophages) with genes that promote host survival should be called ‘mobile’ rather than ‘selfish’.

## Conclusions

*Pseudomonas aeruginosa* is one of the most common opportunistic human pathogens and can establish difficult-to-eradicate infections. Prophages are frequent components of this bacterium’s genome and occasionally enhance its virulence and antibiotic resistance. However, the contributing role of prophages in the adaptation and long-term persistence of the ubiquitous *P. aeruginosa* in its diverse niches is difficult to parse in the face of many possible drivers of fitness. Meanwhile, more and more studies offer evidence that intact prophages contribute to *P. aeruginosa* persistence in the CF airways. For instance, *P. aeruginosa* prophages DMS3 and pp3 were shown to exclude phages that require type IV pilus as a receptor and to assist in host adaptation by promoting biofilm formation, respectively (Li et al. 2017, Shah et al. 2021). In other studies, the acquisition of prophages decreased antibiotic susceptibility and virulence of clinical *P. aeruginosa* and increased biofilm formation (Tariq et al. 2019). Moreover, Burns et al. (2015) demonstrated that in mixed infections PAO1 polylysogens used phage predation to prevail over their isogenic prophage-free competitors. The enhanced competitiveness of PAO1 lysogens against isogenic prophage-free competitors was corroborated by Davies et al. (2016b). The same study also showed that prophages facilitated adaptation in a sputum-like environment either by insertional inactivation of type IV pilus and quorum-sensing-associated genes or by selecting for type IV pilus gene mutations that led to loss of motility, a phenotype found to increase *P. aeruginosa* fitness in the murine airways (Roux et al. 2015).

In this study, we used a unique longitudinal collection of *P. aeruginosa* clinical isolates from different genetic lineages (CTs) evolving in the CF airways niche to evaluate the role of prophages in persistent bacterial infection. Among others, we found that (poly)lysogeny is widespread within our CF *P. aeruginosa* isolates, and that genomes rich in intact prophages are predicted to be less likely to contain active CRISPR-Cas systems. We also described 29 intact prophages with highly diverse genomes. The genomic diversity unlocked by the study is anticipated to aid towards a deeper understanding of the versatility and ecology of *Pseudomonas* prophages and of their interactions with their host. We observed that some of the studied intact prophages induced due to ciprofloxacin, an antibiotic often used for CF patient treatment. In addition to antibiotic-triggered induction, self-induction was observed. Such inducibility patterns could translate into a high availability of free prophages in the CF airways with various implications for the host CT competitiveness, its ease of adaptation and its social interactions. Last but not least, we identified that 14 out of 15 of the inducible prophages displayed a high genomic conservation and repeated detection over a 4 + year time period within the persistent CTs. These two latter features, along with the ease of prophage induction, directly point to their positive selection by the host and suggest that these prophages enhance host fitness and persistence in the CF airways. In the cases of prophages Ostro, Alize, Haboob, Riah, Solano, Shamal, and Lodos, we bioinformatically predicted genes encoding verified AMR and virulence factors as well as genes enhancing bacterial host competitiveness and fitness, which offers additional support to these prophages’ essentiality for the host. Furthermore, the ability of the transposable prophages Bise, Etesian, Gregale, Meltemi, Marin, and Rashabar to randomly integrate into the host genome, thus facilitating rapid adaptation to the CF airways and the varying clinical treatments, likely explains why maintaining their inducibility would be beneficial for the host. Overall, we expect our findings on the genomic diversity and persistence of certain prophages to assist in developing novel antibacterial strategies and diagnostic solutions for *P. aeruginosa* infections.

## Supplementary Material

fnaf017_Supplemental_Files

## Data Availability

Genomes of induced prophages and bacteria, which are first presented in this study have been uploaded to GenBank and their accession numbers can be found in Tables S1 and S5. The genomes and annotation files of the 14 predicted as intact but uninduced prophages are available through Zenodo as indicated in Table S5.
