# Supplementary material for: Pseudomonas aeruginosa maintains an inducible array of novel and diverse prophages over lengthy persistence in cystic fibrosis lungs
Source: FEMS Microbiol Lett. 2025 Jan 31;372:fnaf017. doi: 10.1093/femsle/fnaf017 (PMC11846083; doi:10.1093/femsle/fnaf017)
Supplement: fnaf017_Supplemental_Files [file fnaf017_supplemental_files.zip › Supplementary_Figure_1.docx]

Supplementary Figure 1: Best mapping results of lysate sequencing for the 15 inducible prophages described in this study. Green bundles in the graphs correspond to induced prophages. The location of the bundle in the x axis indicates the prophage’s region of insertion in the host chromosome. The green lines comprising the bundles represent the sum of reads per prophage region and the bundle height shows the approximate depth of coverage of the sequenced prophage genome. Information regarding the name of the sequenced prophage genomes and their host isolate is given below each map. In case of co-induction, differently coloured arrows differentiate between the two induced prophages.


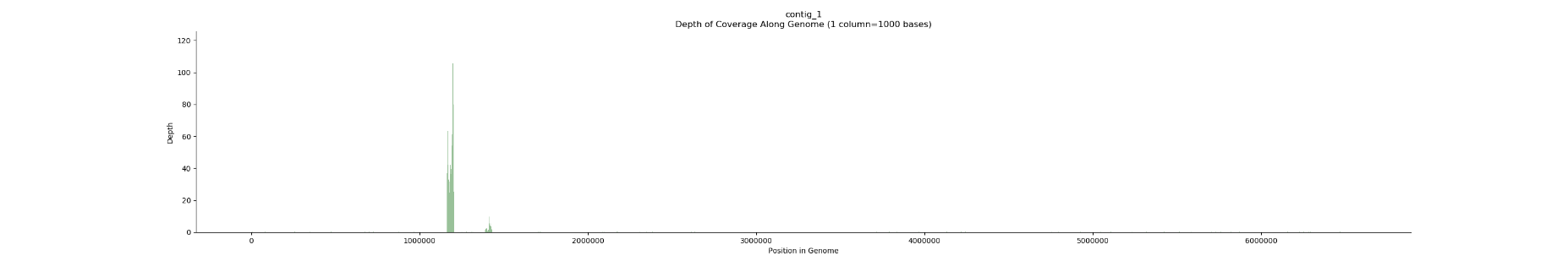


**Pseudomonas phage Turba (host isolate: F038)**


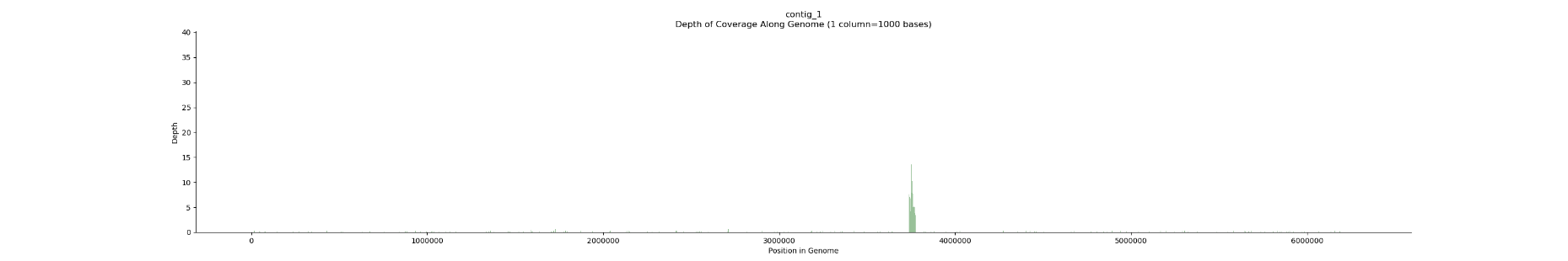

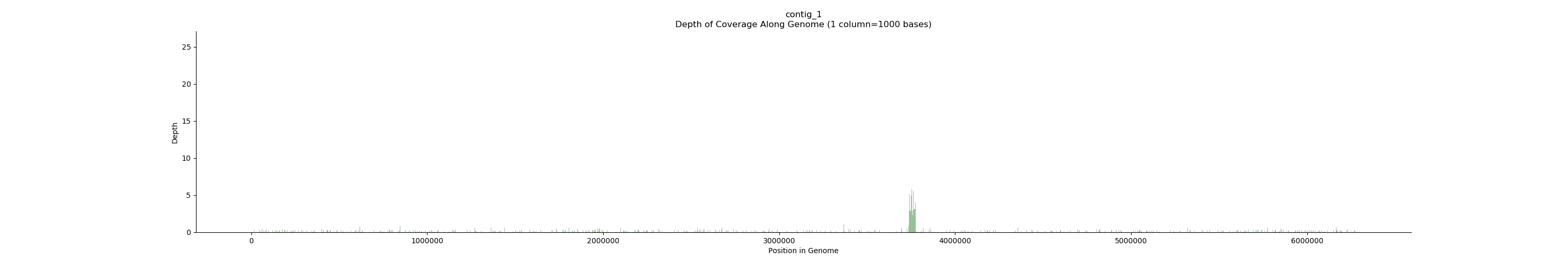

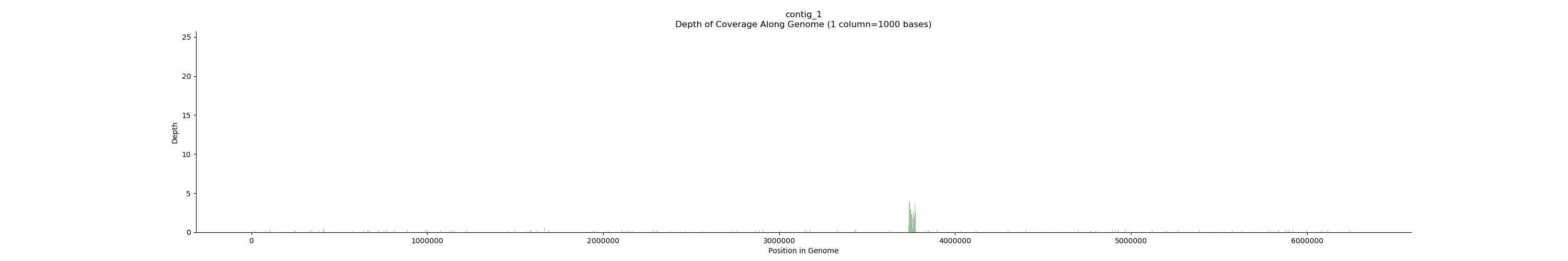


**Pseudomonas phage Bise (host isolate: 199)**


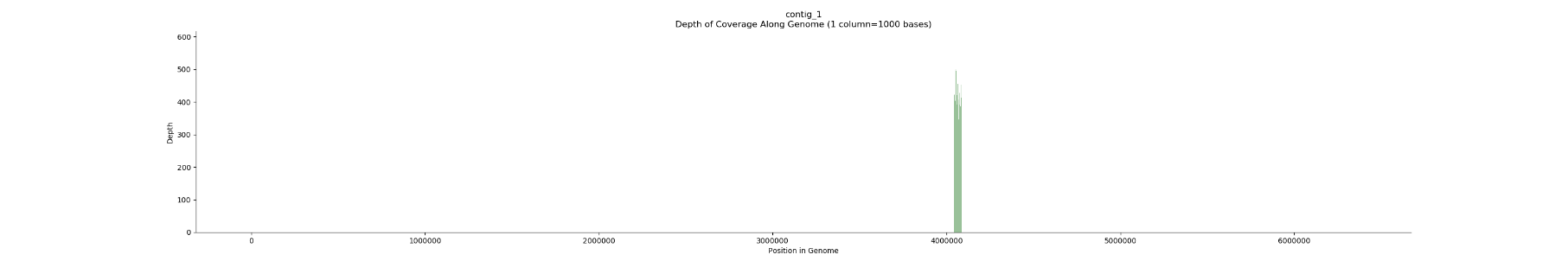


**Pseudomonas phage Riah (host isolate: 20)**


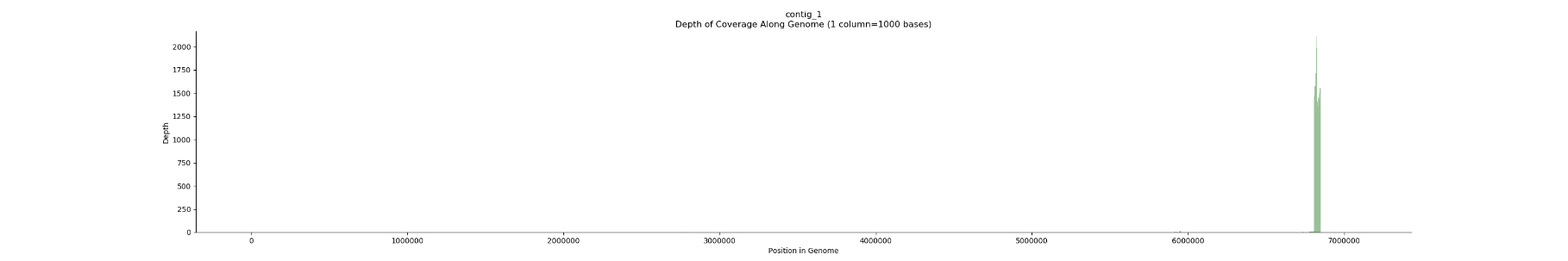


**Pseudomonas phage Shamal (host isolate: F002)**


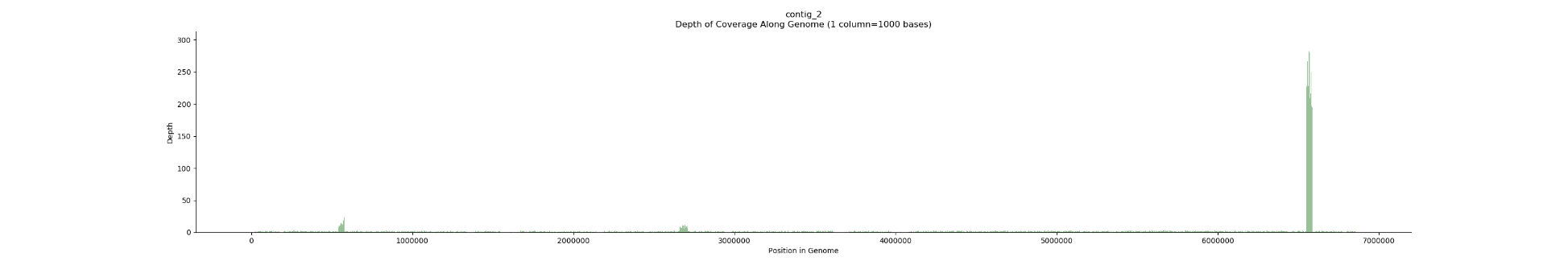

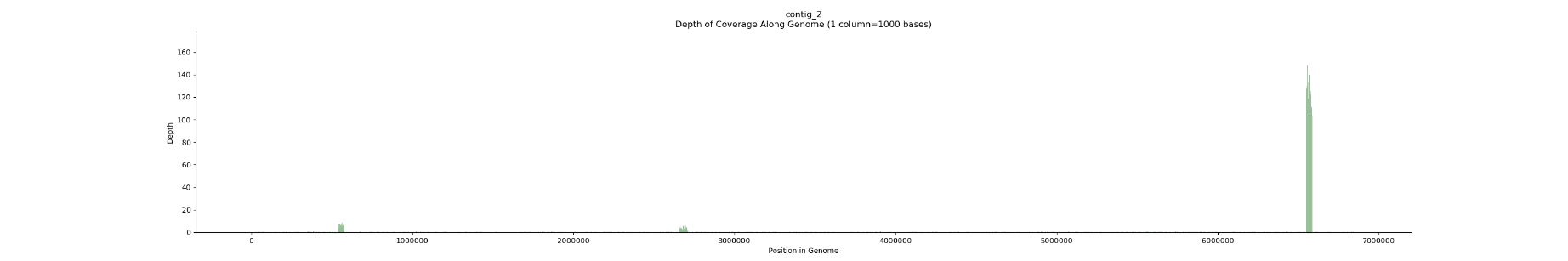


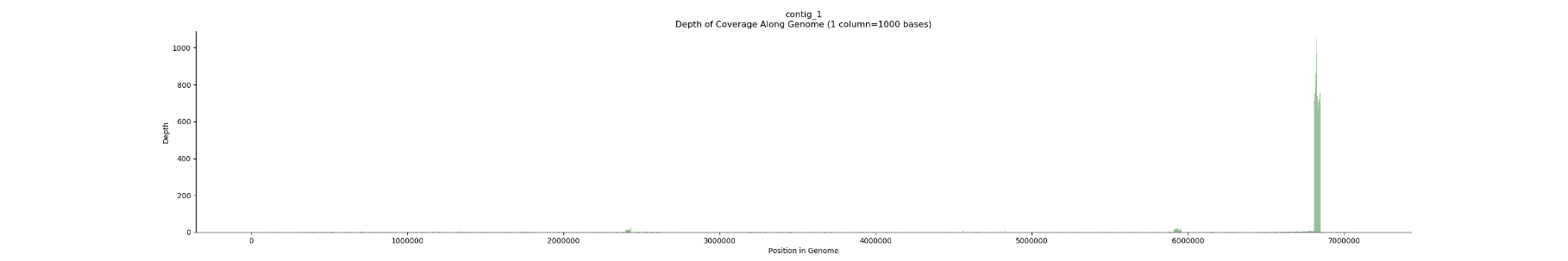


**Pseudomonas phage Gregale (host isolate: F002)**


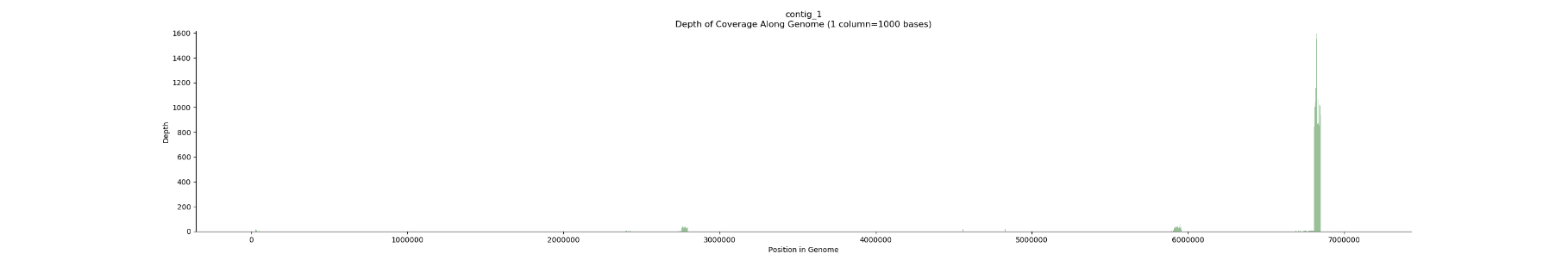


**Pseudomonas phage Marin & Pseudomonas phage Alize (host isolate: F002)**


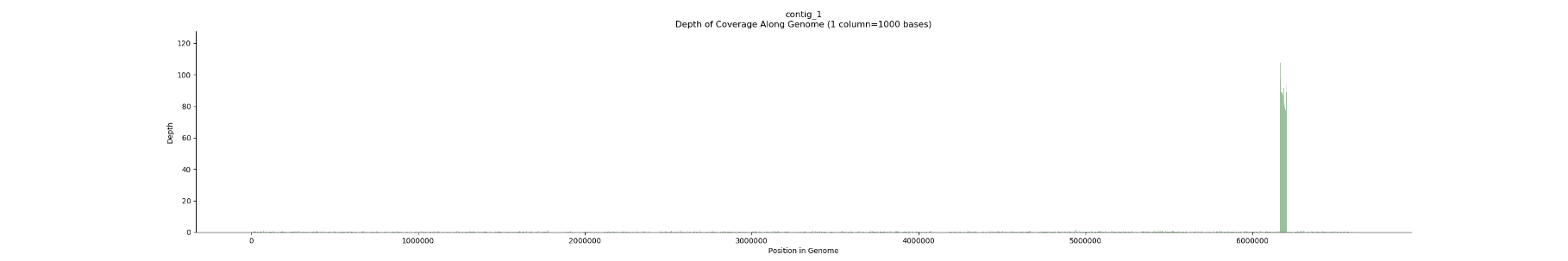


**Pseudomonas phage Rashabar (host isolate: 37)**


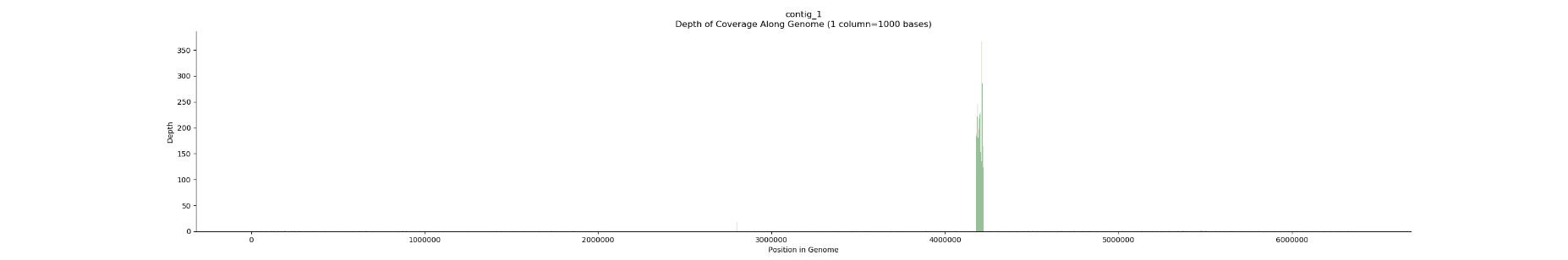


**Pseudomonas phage Lodos (host isolate: 135)**

**Pseudomonas phage Etesian & Pseudomonas phage Haboob (host isolate: 382)**


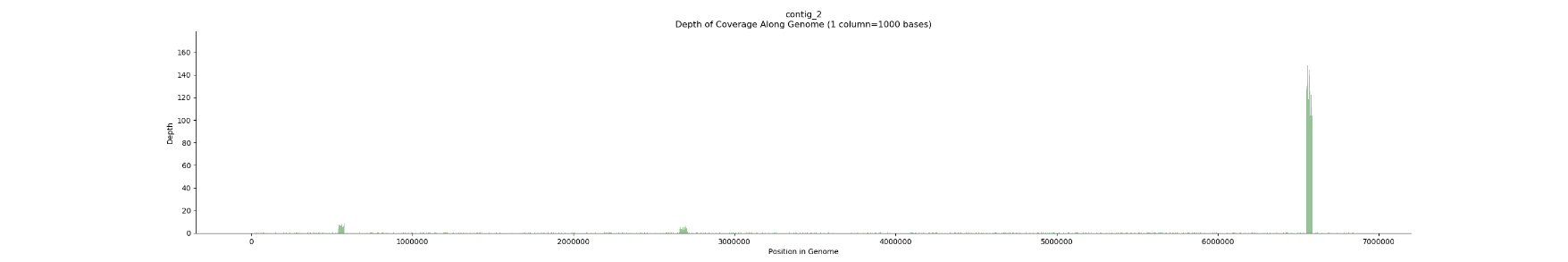


**Pseudomonas phage Ostro (host isolate: 382)**


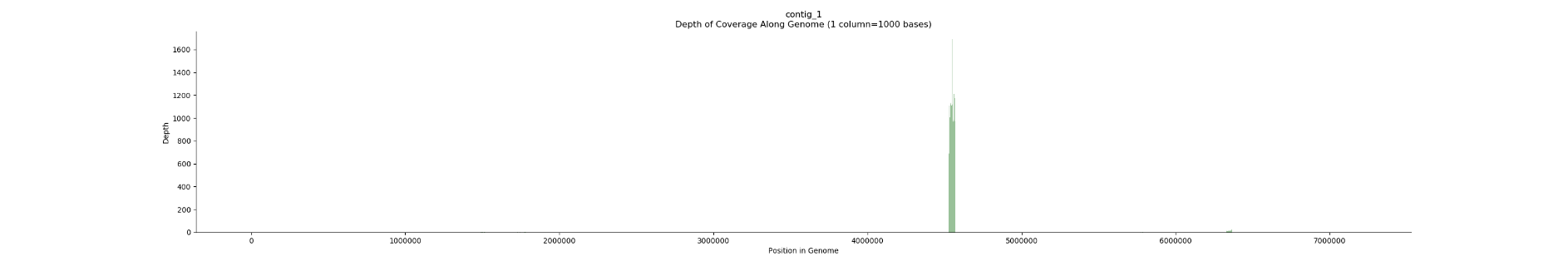


**Pseudomonas phage Solano & Pseudomonas phage Meltemi (host isolate: LRJ32)**


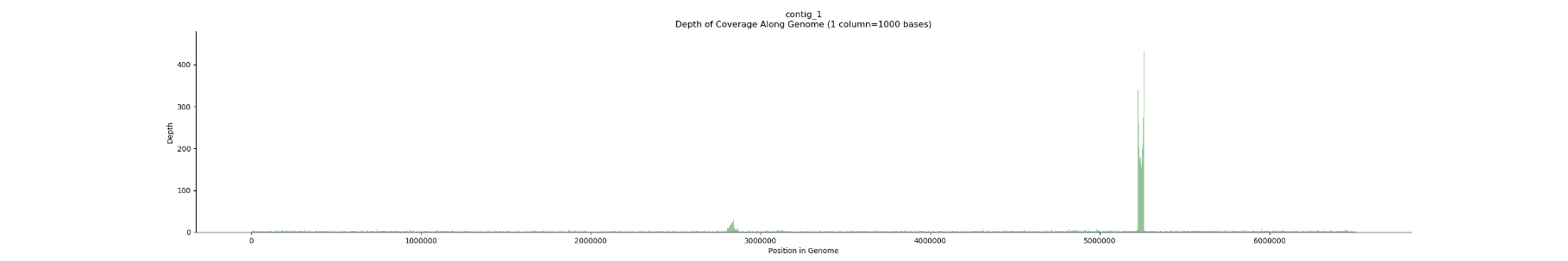


**Pseudomonas phage Sirocco (host isolate: 188)**
