# Supplementary material for: Pseudomonas aeruginosa maintains an inducible array of novel and diverse prophages over lengthy persistence in cystic fibrosis lungs
Source: FEMS Microbiol Lett. 2025 Jan 31;372:fnaf017. doi: 10.1093/femsle/fnaf017 (PMC11846083; doi:10.1093/femsle/fnaf017)

Supplementary Figure 3: BLASTn similarity genomic synteny maps of remaining 13 induced prophages of this study. The maps are designed with Easyfig. A sequenced prophage from an early isolate is compared to its own genome identified in a later longitudinal isolate of the persistent CT.


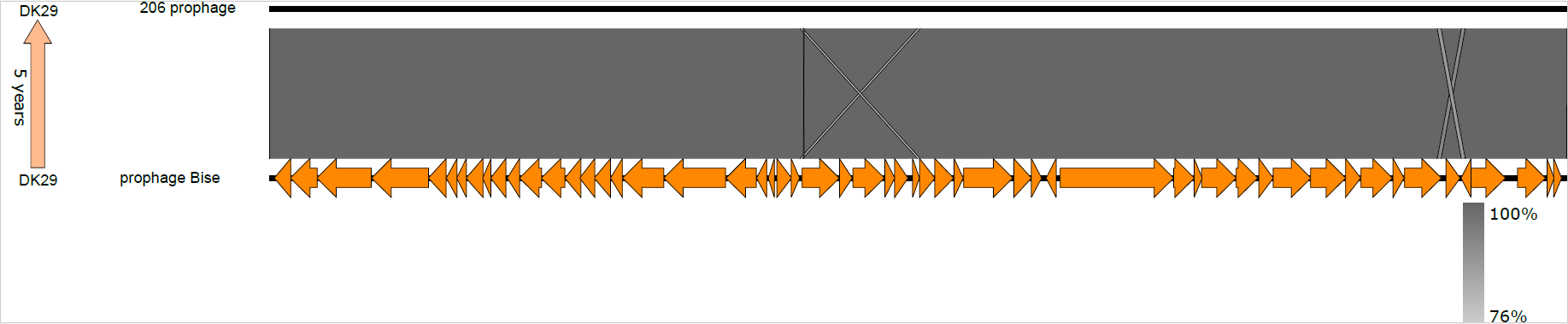


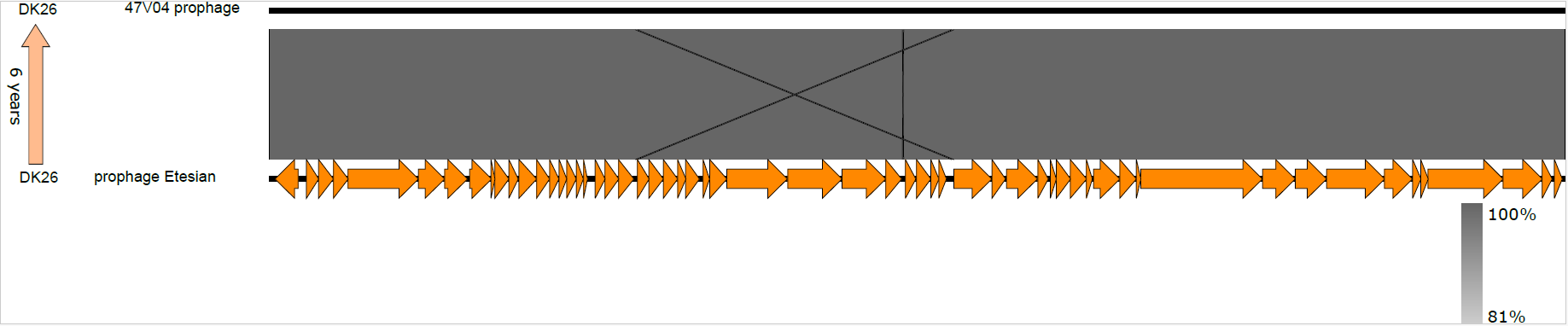


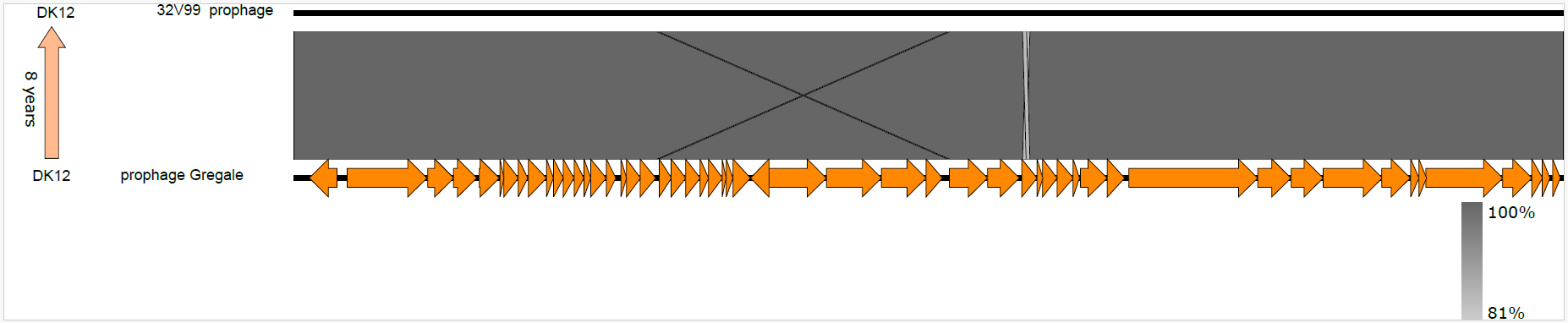


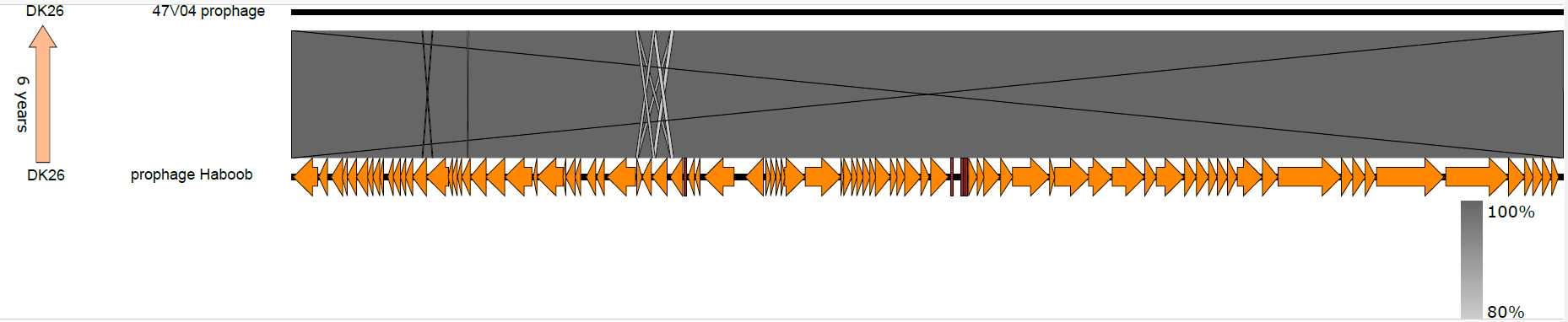


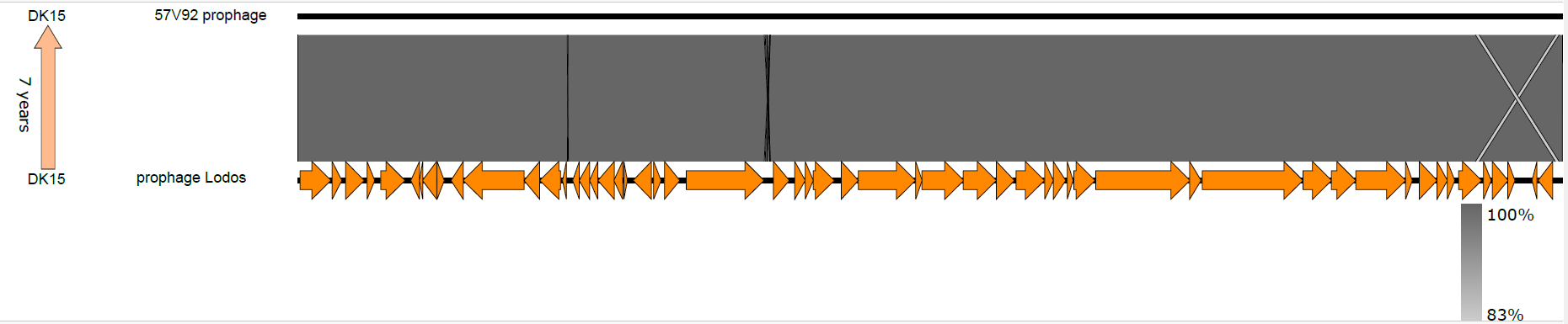


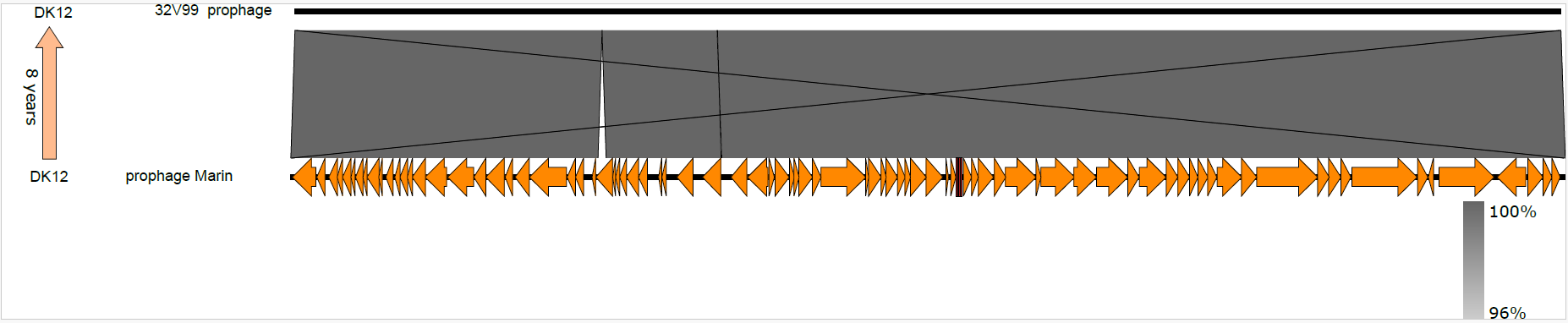


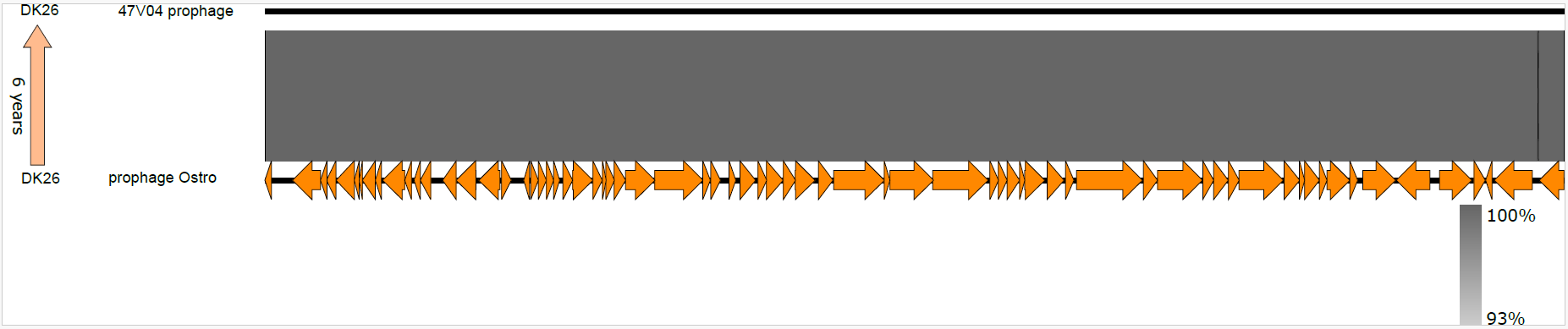


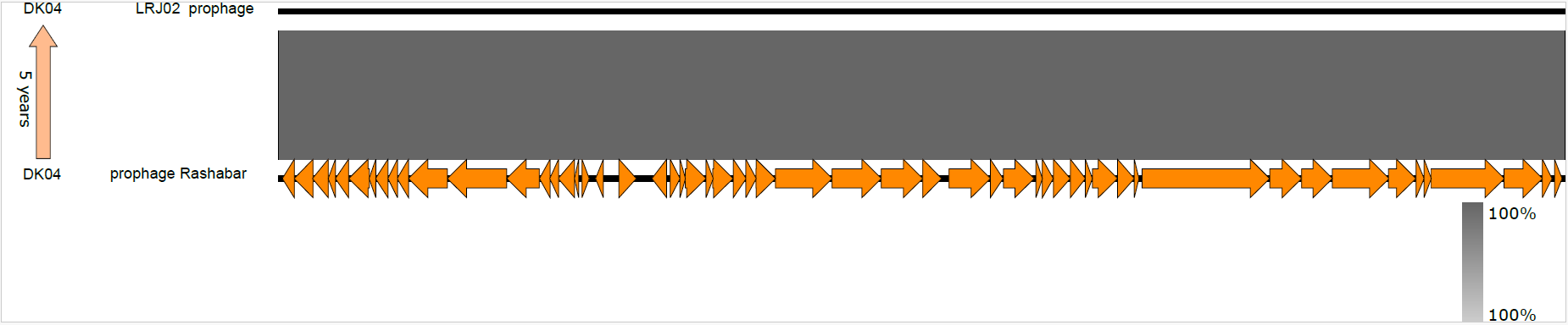


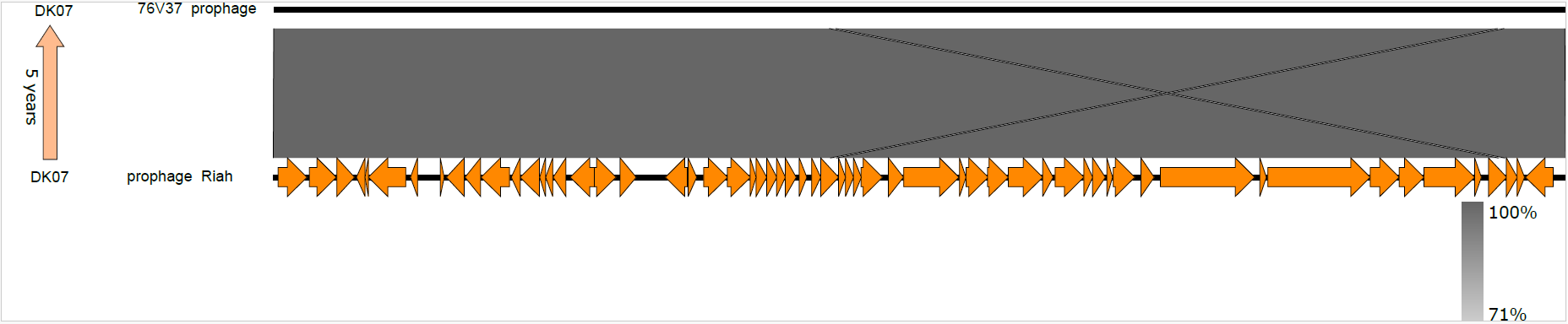


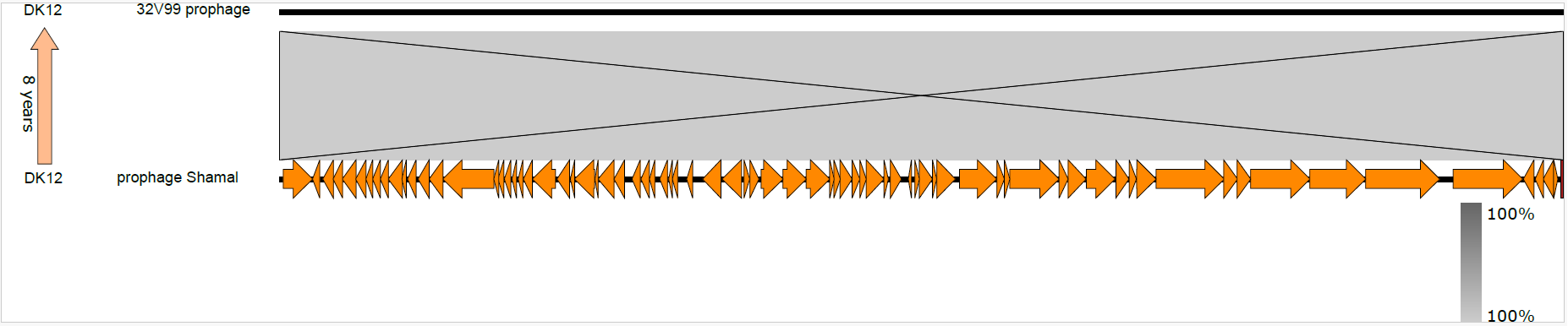


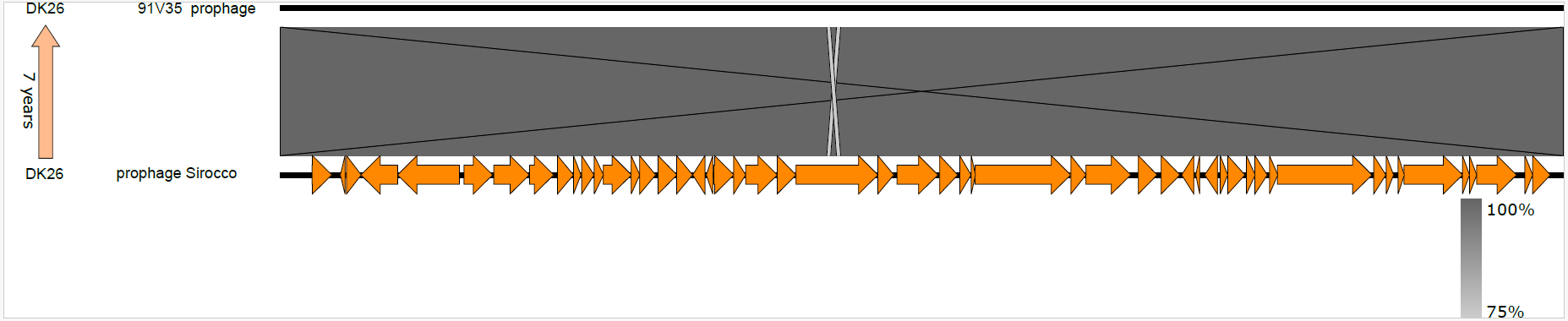


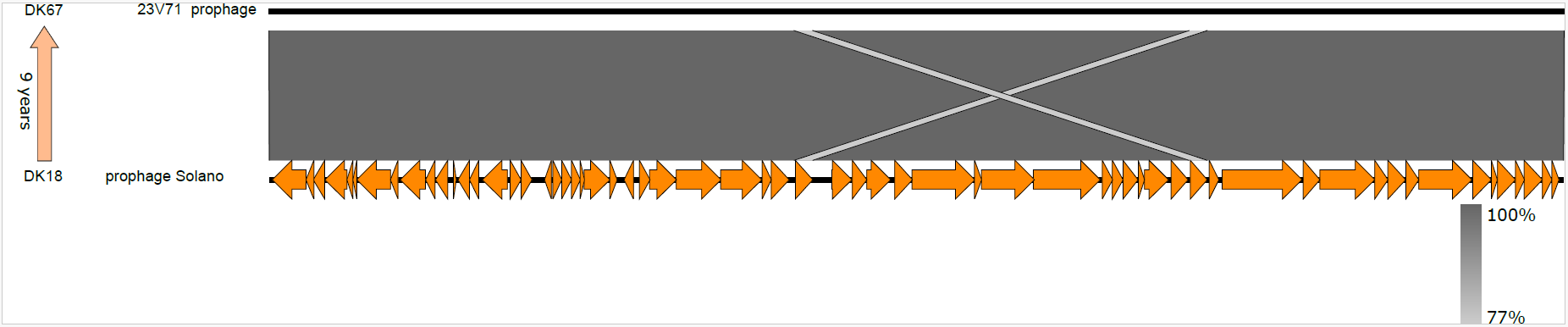


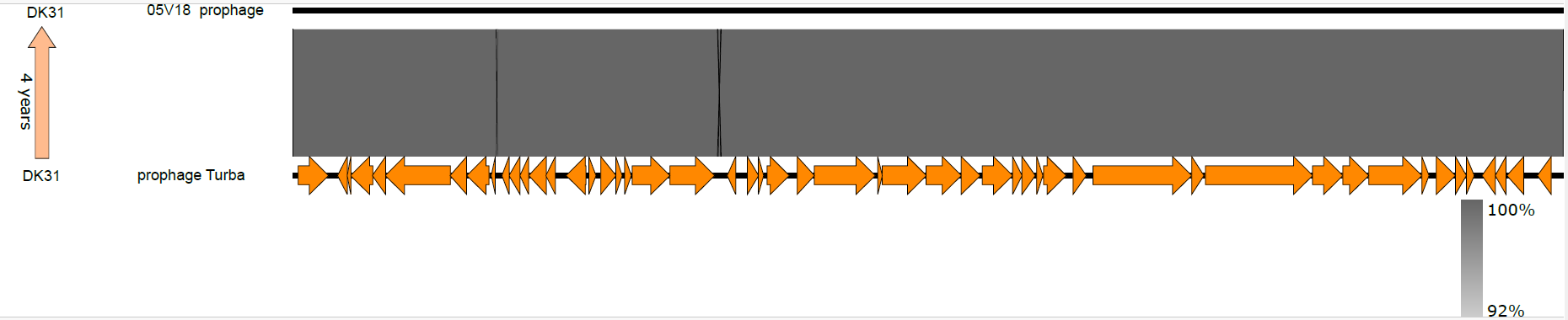

Supplement: fnaf017_Supplemental_Files [file fnaf017_supplemental_files.zip › Supplementary_Figure_3.docx]
