# Supplementary material for: Pseudomonas aeruginosa maintains an inducible array of novel and diverse prophages over lengthy persistence in cystic fibrosis lungs
Source: FEMS Microbiol Lett. 2025 Jan 31;372:fnaf017. doi: 10.1093/femsle/fnaf017 (PMC11846083; doi:10.1093/femsle/fnaf017)
Supplement: fnaf017_Supplemental_Files [file fnaf017_supplemental_files.zip › Supplementary_Information_revised_Captions.docx]

***Pseudomonas aeruginosa* maintains an inducible array of novel and diverse prophages over lengthy persistence in cystic fibrosis lungs**

Supplementary Information Captions

Supplementary Figure 1: Best mapping results of lysate sequencing for the 15 inducible prophages described in this study. Green bundles in the graphs correspond to induced prophages. The location of the bundle in the x axis indicates the prophage’s region of insertion in the host chromosome. The green lines comprising the bundles represent the sum of reads per prophage region and the bundle height shows the approximate depth of coverage of the sequenced prophage genome. Information regarding the name of the sequenced prophage genomes and their host isolate is given below each map. In case of co-induction, differently coloured arrows differentiate between the two induced prophages.

Supplementary Figure 2: Pairwise intergenomic nucleotide-based comparisons of all intact prophages found in this study against a custom, literature-based database of *P. aeruginosa* prophages proven to exist as active particles. The intergenomic similarity scoring and presented heatmap were generated with VIRIDIC.

Supplementary Figure 3: BLASTn similarity genomic synteny maps of remaining 13 induced prophages of this study. The maps are designed with Easyfig. A sequenced prophage from an early isolate is compared to its own genome identified in a later longitudinal isolate of the persistent CT.

Supplementary Table 1: Accession numbers and other information about *P. aeruginosa* clinical isolates used in this study. The symbol ≠ denotes synonymous IDs. For sampling dates, only the year is disclosed to ensure compliance with regulations of the Scientific Ethics Committee at the Capital Region of Denmark (Region Hovedstaden) and protection of patient personal data. Column G presents information on how many longitudinally persistent and induced prophages were counted per isolate out of the total number of such prophages found within each patient case (column D). Columns H-V give per isolate (column B) details on the presence (1) or absence (0) of each longitudinally persistent prophage found in the patient (column D) where the isolate in question originated from. Columns W-AJ give the same information as before but for each predicted as intact but uninduced prophage. When a prophage was found in 80% of the dates corresponding to the persistent CT it was deemed longitudinally persistent.

Supplementary Table 2: Rigshospitalet records of E-test-based ciprofloxacin minimum inhibitory concentrations (MIC) for all 12 *P. aeruginosa* isolates used in this study.

Supplementary Table 3: Literature-based database of all *P. aeruginosa* prophages proven to exist as active particles. Last update June 1st 2023.

Supplementary Table 4: Comparison of the accuracy of Prophage Hunter and PHASTER in predicting the 29 induced and likely intact but uninduced prophages of this study.

Supplementary Table 5: Accession numbers and other information about the 29 prophages identified in this study. The genomes of all 14 likely intact but uninduced prophages have been deposited at Zenodo together with their annotation files.

Supplementary Table 6: BLASTn comparison results of the 29 prophages of this study to the viral nucleotide collection database of NCBI (taxid:10239). As most related phage genome, we list the complete phage genome with the highest %similarity (query cover x percentage identity) result. Whenever the bacterial host of the most related phage could be identified from the literature, that was always a bacterium originating from clinical settings.
